# How peer influence shapes value computation in moral decision-making

**DOI:** 10.1016/j.cognition.2021.104641

**Published:** 2021-06

**Authors:** Hongbo Yu, Jenifer Z. Siegel, John A. Clithero, Molly J. Crockett

**Affiliations:** a Department of Psychology, Yale University, New Haven, CT, USA; b Lundquist College of Business, University of Oregon, Eugene, Oregon, USA

**Keywords:** Moral decision-making, Social influence, Goal, Drift diffusion model, Bayesian hierarchical modeling

## Abstract

Moral behavior is susceptible to peer influence. How does information from peers influence moral preferences? We used drift-diffusion modeling to show that peer influence changes the value of moral behavior by prioritizing the choice attributes that align with peers' goals. Study 1 (*N* = 100; preregistered) showed that participants accurately inferred the goals of prosocial and antisocial peers when observing their moral decisions. In Study 2 (*N* = 68), participants made moral decisions before and after observing the decisions of a prosocial or antisocial peer. Peer observation caused participants' own preferences to resemble those of their peers. This peer influence effect on value computation manifested as an increased weight on choice attributes promoting the peers' goals that occurred independently from peer influence on initial choice bias. Participants' self-reported awareness of influence tracked more closely with computational measures of prosocial than antisocial influence. Our findings have implications for bolstering and blocking the effects of prosocial and antisocial influence on moral behavior.

## 1 Introduction

Moral behavior is contagious. Observing generous, cooperative or helpful behavior in peers encourages people to adopt similar behaviors themselves (Dimant, 2019; Jung, Seo, Han, Henderson, & Patall, 2020; Nook, Ong, Morelli, Mitchell, & Zaki, 2016), and people are more likely to lie, steal, punish and harm others when their peers do the same (Bandura, Ross, & Ross, 1961; Chierchia, Pi-Sunyer, & Blakemore, 2020; Dimant, 2019; Fabbri & Carbonara, 2017; FeldmanHall, Otto, & Phelps, 2018; Gino, Ayal, & Ariely, 2009; Son, Bhandari, & FeldmanHall, 2019). Although peer influence on moral behavior (or moral influence) is well-documented, several open questions remain. First, it is unknown how information about peers' behavior affects the computations that guide moral decision-making. Second, it is unclear to what extent people can accurately report peer influence on their decisions. Here, we address these questions by building on insights into the computational processes guiding value-based decision-making.

Recent work indicates that decisions are made by comparing choice options in terms of their subjective values, which are integrated over the options' attributes, such as healthfulness, tastiness, and price of snacks (Hare, Malmaud, & Rangel, 2011; Krajbich, Armel, & Rangel, 2010; Maier, Beharelle, Polanía, Ruff, & Hare, 2020; Sullivan, Hutcherson, Harris, & Rangel, 2015). The way these attributes are integrated is highly sensitive to a decision-maker's goals, here conceptualized as a desired end-point state of the decision-maker (Fishbach & Ferguson, 2007). Specifically, goals influence decision-making by prioritizing attributes that are consistent with the current goal (Rangel & Hare, 2010). For example, when choosing between a tasty, unhealthy snack and a healthier but less tasty snack, having a goal to lose weight increases the weight on healthiness in the computation of subjective values (Hare et al., 2011). Likewise, when deciding how to allocate money between oneself and another person, having a goal to consider the ethical implications of choices increases the weight on others' payoffs in subjective value computation (Tusche & Hutcherson, 2018).

Crucially, one person's goals can influence the goals, preference and choices of other people (Aarts, Gollwitzer, & Hassin, 2004; Dijksterhuis & Aarts, 2010). For instance, learning about someone with a goal to earn money increases the motivation to earn money oneself (Aarts et al., 2004; Vohs, Mead, & Goode, 2008). One interpretation is that using a goal representation to understand another person's behavior makes that goal more accessible in the perceiver's own subsequent behaviors (Custers & Aarts, 2007). If inferring the goal-directed preferences of others makes those goals more salient for oneself, this could increase the weights of goal-consistent choice attributes – just as activating health or ethical goals increases the weight of health and ethical attributes during individual decision-making (Hare et al., 2011; Tusche & Hutcherson, 2018). As people are more likely to adopt the goals and preferences of similar others (Loersch, Aarts, Payne, & Jefferis, 2008), we might expect prioritization of inferred goal-consistent attributes to be stronger when peers are more similar to oneself. This process is distinct from mere imitative behavior; while an imitative account of peer influence would predict that individuals will be biased toward simply copying the harmful or helpful behaviors of observed peers (Chartrand & Bargh, 1999; Chartrand & Lakin, 2013; Heyes, 2011), a valuation account of peer influence would predict that peer influence changes the subjective value of moral behaviors, over and above any imitative effects.

We addressed these questions in a setting where participants made moral decisions about whether to profit from inflicting pain on a stranger (Crockett, Siegel, Kurth-Nelson, Dayan, & Dolan, 2017). In Study 1 (*N* = 100), we demonstrated that people could accurately predict peers' goal-directed choices and readily infer prosocial and antisocial preferences when observing peers make such decisions. In Study 2 (*N* = 68), we modeled the effects of peer influence on moral decisions using a multi-attribute extension of the drift-diffusion model (DDM) (Ratcliff & McKoon, 2008; Wiecki, Sofer, & Frank, 2013). In this model, choice options are compared in terms of their subjective values that are integrated over multiple attributes (in our case, profit for self and pain for another). Over time, value accumulates into a decision variable that represents accumulated evidence in favor of one option over another. A choice is made when the decision variable passes a threshold for one of the choice options.

Several recent studies use the drift diffusion model (DDM) to investigate the cognitive mechanisms underlying conformity (Germar, Schlemmer, Krug, Voss, & Mojzisch, 2014; Son et al., 2019; Tump, Pleskac, & Kurvers, 2020). For example, Germar et al. (2014) manipulated the majority choice in a perceptual binary decision task and found that the social consensus information modulated participants' drift rate toward the majority option but not the decision threshold. More recently, Tump et al. (2020) used a more sophisticated experimental design and computational model to demonstrate how in a sequential decision-making context, individuals dynamically integrate perceptual and social information over time. However, neither of these studies directly examined moral decision-making, and therefore it remains unclear whether moral decision-making may employ the same or a different set of computational processes compared with non-moral decision-making (e.g., Cushman, 2013; Lockwood, Apps, & Chang, 2020). More relevant to the current research, Son et al. (2019) investigated how consensus in punishment decision of a group, either as a group of victims or as a group of jurors, influences individual members' punishment decisions. They found that, although both the victims and the jurors are swayed by their groups' consensus in punishment decision, the jurors' evidence accumulation (i.e., drift rate) was more sensitive to the severity of the crime and less influenced by the group's decision. All three of these studies, however, investigated one form of social influence, namely, conformity to a group's consensus. There are other forms of social influence, such as compliance and emulation, that may rely on different cognitive mechanisms (Cialdini & Goldstein, 2004; Kristjánsson, 2006). Specifically, the motivations that drive people to imitate the behaviors of a moral exemplar or role model may be different compared to the motivations that drive people to follow the statistical majority of a group. Moreover, the impact of similarity between oneself and one's role model on the extent of moral emulation is not well understood.

In the current studies, moral influence effects could manifest in multiple ways. First, it is possible that observers simply imitate the peers' behaviors unconditionally, such that observing a prosocial peer would make participants more biased toward minimizing pain, while observing an antisocial peer would make participants more biased toward maximizing profit, regardless of the amount of pain or profit. Second, we predicted that observing a prosocial peer who prioritizes minimizing the pain of others over maximizing profit for themselves would increase the (negative) impact of pain on value accumulation, while observing an antisocial peer who prioritizes maximizing profit over minimizing the pain of others would increase the (positive) impact of profit on value accumulation. Our DDM framework allowed us to identify peer influence effects on both choice biases and valuation processes, which are separately parameterized in the model.

Our modeling approach also allowed us to test how accurately people detect peer influence on their own decisions. Some work suggests people are often unaware of peer influence effects (Bargh, Gollwitzer, Lee-Chai, Barndollar, & Trötschel, 2001; Nolan, Schultz, Cialdini, Goldstein, & Griskevicius, 2008). Yet, there is evidence that people have accurate metacognitive awareness of many aspects of their own decision-making (for a review, see Fleming & Dolan, 2012). One possible explanation for this discrepancy is that past work has not systematically distinguished awareness of prosocial and antisocial influence. Because people are strongly motivated to preserve a moral self-image (Mazar, Amir, & Ariely, 2008), they may be less willing or able to recognize or report antisocial influence than prosocial influence. We tested this possibility by comparing participants' self-reported awareness of peer influence with our computational measures of actual influence, predicting that self-reports would be more aligned with computational measures for prosocial relative to antisocial influence.

In summary, our study advances the understanding of moral influence in four ways: (i) by investigating how privately learning the moral preferences of a peer influences the valuation of one's own moral decision-making; (ii) by examining whether prosocial and antisocial peers exert influences on the same or different components of the moral valuation process; (iii) by studying how objective and subjective similarity between oneself and one's role model affects the extent and nature of influence; and (iv) by elucidating the relationship between awareness of and actual peer influence .

## 2 Study 1

In Study 1 (pre-registered: https://aspredicted.org/8xw3q.pdf), we tested the hypothesis that participants would be able to accurately infer the goals of prosocial and antisocial peers when observing them complete a moral decision-making task.

### 2.1 Materials and methods

#### 2.1.1 Participants

One hundred U.K residents (50 female, 50 male; mean age 24.6 ± 3.1 years) were recruited using the online platform Prolific (www.prolific.ac). The sample size of Study 1 was determined by a power analysis prior to data collection, which can be found in the pre-registration (https://aspredicted.org/8xw3q.pdf). All participants provided written consent prior to participation. The study was approved by Yale Human Subjects Committee (2000022385). Participants were incentivized for accurate prediction. Specifically, participants whose prediction accuracy was higher than 80% would receive a monetary bonus of $0.3, those whose prediction accuracy was between 65% and 80% would receive a monetary bonus of $0.2, and finally those whose prediction accuracy was between 51% and 65% would receive a monetary bonus of $0.1.

#### 2.1.2 Procedure

**Fig. 1:**
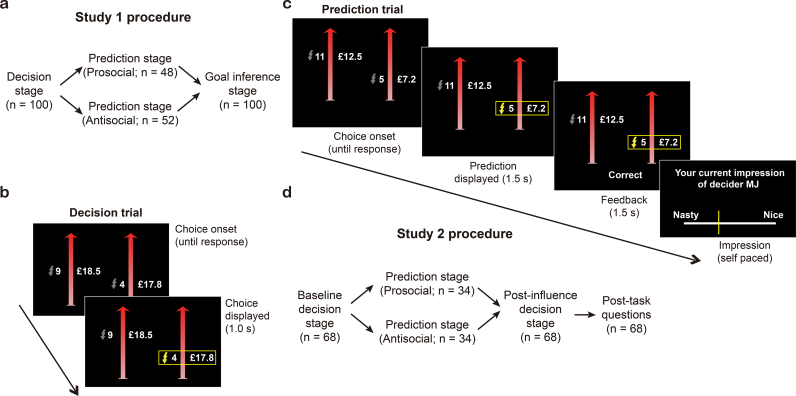
Experimental design and procedure. (a) Overview of the procedure of Study 1. In the Decision stage, all participants completed a moral decision-making task (as in **b**). Subsequently in the Prediction stage, participants were randomly assigned to predict the choices of a prosocial or antisocial peer in the same moral decision-making task (as in **c**). Finally, they were asked to infer the goal of the peer they predicted. (b) In a moral decision-making trial, participants made (hypothetically in Study 1, for real in Study 2) a series of choices between a harmful option that entailed more money for themselves and more shocks for an anonymous ‘receiver’, and a helpful option that entailed less money for themselves and fewer shocks for the receiver. (c) In a prediction trial, participants predicted what a peer would choose on a given trial and received feedback about their prediction at the end of each trial. Participants' impressions of the peer's character were measured periodically during the prediction stage. (d) Overview of the moral influence paradigm of Study 2. The first two stages (baseline decision stage and prediction stage) were the same as in Study 1. In the third, post-influence decision stage, participants completed the moral decision-making task again (as in **b**). After that, they would answer questions about their awareness of behavioral changes in the post-influence relative to the baseline decision stages.

The task was administered online and consisted of three stages (Fig. 1a). In the decision stage, participants adopted the role of ‘decider’ in a task where they made a series of 20 hypothetical decisions that involved choosing between a harmful option and a helpful option (Fig. 1b). The harmful option was financially more beneficial to the decider, but delivered more painful electric shocks to another person (the ‘receiver’). The relative positions of harmful and helpful options on the screen were randomized throughout the experiment.

In the prediction stage (Fig. 1c**)**, participants predicted a series of 50 decisions made by a peer who had previously participated in the role of decider. In a between-subjects design, participants were randomly assigned to predict the choices of one of two peers who significantly differed in their moral preferences (i.e., their preferences toward harming the receiver): the ‘antisocial peer’ required significantly less money to increase electric shocks to the receiver and therefore was more harmful than the ‘prosocial peer’. Forty-eight participants (25 female) and fifty-two participants (25 female) were assigned to predict the choices of the prosocial peer and the antisocial peer, respectively. On each trial, participants first saw the choice options that the peer was faced with, and subsequently predicted which option the peer chose. Finally, they received feedback as to whether their prediction was correct or incorrect (cf. Siegel, Mathys, Rutledge, & Crockett, 2018). They were explicitly incentivized to be as accurate as possible in their predictions. Participants also periodically (every 3 trials) indicated their impressions of the peer's moral character on a scale ranging from 0 (nasty) to 1 (nice). Before making any predictions, participants indicated how nasty or nice they expected the peer would be, which provided an indication of participants' prior expectations about people's moral character. Finally, in the goal inference stage, we asked participants to make explicit inferences about the goals of the peer whose choices they had just predicted. Participants evaluated the extent to which they agreed or disagreed with the following statements about the peer's goals on a 7-point Likert sale (1 = strongly disagree, 4 = neither agree nor disagree, 7 = strongly agree):

(a). The decider's main goal was to earn as much money for themselves as possible.

(b). The decider's main goal was to avoid as many shocks to the receiver as possible.

Statement (a) is a money-maximizing goal while statement (b) is a shock-minimizing goal. Next, participants were asked to make a forced binary choice between these two alternative goals as to which one better described the observed peer's goal.

#### 2.1.3 Determination of trial sets: decision task

We created a set of 20 trials, each containing a pair of options, one of which (the harmful option) contained more money for the decider and more electric shocks to the receiver than the other (the helpful option). Each trial was characterized by a unique combination of shock difference (Δs) and money difference (Δm) between the two options. We define trial κ as Δm/(Δs + Δm) for each trial. Trial κ reflects the exchange rate between money and shocks on a given trial (Crockett et al., 2017): the higher this value is, the more profitable the harmful option is for a given amount of increase in shocks. Therefore, a decider will be more likely to choose the harmful option as a trial's κ value approaches 1. The trial κ value where a decider is indifferent to the two options is defined as the decider's harm aversion.

In the decision task, we set the trial κ to be evenly distributed between 0.05 and 0.95. To do that, for each κ value we generated 10,000 random pairs of positive shock differences Δs (from 1 to 19) and positive money difference Δm (from £0.05 to £19.95) and selected the pair of [Δs, Δm] closest to that κ value. Next, these pairs [Δs, Δm] were transformed into binary choices comprising of an option with a lower number of shocks and amount of money (i.e., the helpful option) and an option with a higher number of shocks and amount of money (i.e., the harmful option). The money for the helpful option (*m*_help_) was a positive number between 0.05 and 19.95 (rounded to the nearest 20th), randomly drawn from a uniform discrete distribution with the constraint that 0.10 ≤ *m*_help_ + Δm ≤ 20.00. Similarly, the shock for the helpful option (*s*_help_) was a positive integer between 1 and 19, randomly drawn from a uniform discrete distribution with the constraint that 2 ≤ *s*_help_ + Δs ≤ 20. Once *m*_help_ and *s*_help_ were determined, *m*_harm_ and *s*_harm_ thus followed: *m*_harm_ ≡ *m*_help_ + Δm, *s*_harm_ ≡ *s*_help_ + Δs.

#### 2.1.4 Determination of trial sets: prediction task

The trial sets for the prediction task were created in two steps: first to create 50 binary choices based on 50 combinations of [Δs, Δm], second to determine the simulated peers' decisions in those binary choices. The peers' decisions were characterized by a utility model that quantifies the exchange rate between money and pain, as defined earlier. This model describes the difference in subjective value (∆V) for choosing the harmful option over the helpful option as a function of the differences in money (∆m) and shocks (∆s) between the harmful and helpful options scaled by a decider's (i.e., peer's) harm aversion parameter (κ).

(1) $$\text{∆V}=\left(1-\kappa \right)\;\text{∆m}–\kappa \;\text{∆s}$$

The harm aversion parameter κ in this model characterizes the relative weights of the differences in money (∆m) and shocks (∆s) between the harmful and helpful options. When κ = 0, deciders will accept any number of shocks to gain money. As κ approaches 1, deciders become highly harm averse and will refuse to deliver an additional shock even for a huge amount of money. Mathematically, a decider harm aversion parameter (κ) is equivalent to the decider's indifference point regarding the harmful and helpful options in trial κ space.

In the prediction task, the prosocial peer's harm aversion (κ) was 0.8 and the antisocial peer's harm aversion was 0.2. This way, the prosocial and antisocial peers would substantially differ in their preferences toward harming the receiver. A κ of 0.8 means that the decider is willing to sacrifice up to $4 in order to reduce the receiver's shocks by 1 shock, while a κ of 0.2 means that the decider won't sacrifice more than $0.25 in order to reduce the receiver's shocks by 1 shock. The self-reported results of both Study 1 and Study 2 showed that participants clearly made divergent moral inferences about the agents with different harm aversion preferences, as indicated by their moral impression ratings and person perception ratings (e.g., likeability, trustworthy, etc.).

We created more trials where the trial κ was close to the respective peer's harm aversion. Choices in those trials are more informative or ‘diagnostic’ of the decider's underlying preference. To do so, for the trial sets for the prosocial peer, we first created a set of 49 trials, in 41 of which trial κ were randomly drawn from a uniform distribution within the range of 0.05–0.95, whereas in the remaining 8 trials the values of trial κ were randomly drawn from a normal distribution around κ = 0.8, which was the simulated prosocial peer's harm aversion (mean = 0.8, s.d. = 0.1). Next, we created a set of 49 matched trials around the antisocial peer's indifference point by subtracting each trial κ value of the prosocial peer's sequence from 1 (i.e., a mirrored sequence). These pairs comprised the second through the 50th trials of the sequences, while the κ value of the first trial was fixed to 0.5. Using the same procedure as described above (see **2.1.3. Determination of trial sets: decision task** above), we converted the sequences of trial κ first into pairs of shock and money differences [Δs, Δm] and then into sequences of binary choices (*m*_harm_ / *s*_harm_ and *m*_help_ / *s*_help_).

Once the trial sets were determined, we next simulated the peers' choices. Given the value of Δs and Δm, and the peer's κ, ∆V of each trial can be computed based on (Eq. 1). A softmax function was used to transform ∆V into a probability of choosing the harmful option over the helpful option, *P*(harm):

(2) $$P\left(\mathrm{harm}\right)=\frac{1}{1+e^{-\beta \times \mathrm{\Delta V}}}$$

where β determines the shape of the sigmoid function. We set β to 100 in order to facilitate participants' learning, as previous work using this task has shown that learning is slower when agent choices are noisier (Study 3, Siegel et al., 2018). Although the relationship between agent unpredictability (or choice noisiness) and moral inference is an interesting question in its own right, it is beyond the scope of the current study. Here, our goal was to make sure the participants clearly learn the preference of the peers, and to examine how such moral inference influences the participants own subsequent decision-making.

We converted the probability of choosing the more harmful option, *P*(harm), into a binary choice, u, using the following equation,

(3) u=1,xrand<Pharm0,xrand≥Pharm

where x_rand_ is a random number between 0 and 1.

### 2.2 Results

#### 2.2.1 Prediction accuracy and impressions of peers

Prediction accuracy data indicated that participants were able to predict peers' choices with an overall accuracy of 78% by the final 10 trials of the prediction stage. Accuracy was higher for participants randomized to predict the choices of an antisocial peer (M ± s.e.m. = 83% ± 2%) than the prosocial peer (M ± s.e.m. = 73% ± 2%; Mann-Whitney *U* test: *z* = 3.25 *P* = 0.001, effect size *r* = 0.33). These findings are consistent with previous studies showing that people are more accurate at predicting the choices of an antisocial agent than a prosocial agent (Siegel et al., 2018).

Subjective impression ratings also indicated that participants distinguished between peers on the basis of moral character. Prior impressions did not differ between groups (antisocial group: M ± s.e.m. = 0.49 ± 0.03; prosocial group: M ± s.e.m. = 0.51 ± 0.03; Mann-Whitney *U* test: *z* = 0.19, *P* = 0.847, effect size *r* = 0.02). For the remaining impression ratings, we used linear mixed-effects models to estimate how impressions differed between the two peers and how they evolved over time. Specifically, group, trial number (i.e., time into the task), and their interaction were included as fixed effects, and trial number was included as random slope nested on participant. We justify our choice of models for the prediction task by using the same models as in previously published work (Siegel et al., 2018). Here, we used an established task (Siegel et al., 2018), measuring the same variables as in this previously published work for completeness, even though many of these variables are not relevant to the present research question. This analysis revealed that, on average, impressions of the prosocial peer were significantly more positive than the impressions of the antisocial peer (*B* = 0.20 ± 0.04, CI = [0.13, 0.28], *t* = 5.43, *P* < 0.001), indicating that our manipulation of the peer's preference successfully induced a significant difference in participants' impressions of moral character. Over time, the difference in impression of the two peers increased, as indicated by a significant group-by-time interaction (*B* = 0.005 ± 0.03, CI = [0.000, 0.011], *t* = 2.02, *P* = 0.047).

**Fig. 2:**
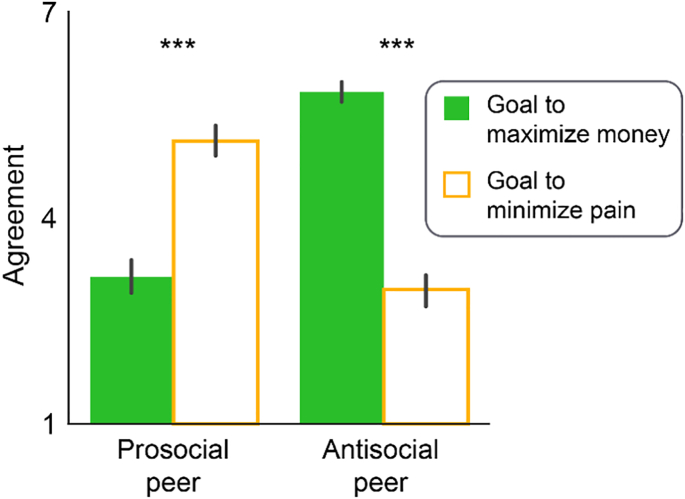
Explicit inference of prosocial and antisocial goals for the prosocial and antisocial peers. Error bars indicate s.e.m. ***: *p* < 0.001. Wilcoxon signed-rank test was used to determine significance. *N* = 48 for the Prosocial peer, *N* = 52 for the Antisocial peer.

Participants' ratings of the observed peer's goals indicated that participants accurately inferred the goals of their peers (Fig. 2). Specifically, participants believed that the antisocial peer was more motivated to maximize their own money (5.83 ± 0.22) than to minimize shocks to the receiver shock (2.96 ± 0.23; Wilcoxon signed-rank test: *z* = 5.59, *P* < 0.001, effect size *r* = 0.78). Moreover, the mean agreement rating for the money-maximizing goal was significantly above the midpoint (i.e., 4) of the scale (one-sample Wilcoxon signed-rank test: *z* = 5.86, *P* < 0.001, effect size *r* = 0.81), while the mean agreement rating for the shock-minimizing goal was significantly below the midpoint (Wilcoxon signed-rank test: *z* = −4.04, *P* < 0.001, effect size *r* = 0.56). In contrast, participants believed the prosocial peer was more motivated to minimize shocks to the receiver (5.12 ± 0.22) than to maximize their own money (3.15 ± 0.21; Wilcoxon signed-rank test: *z* = 3.98, *P* < 0.001, effect size *r* = 0.57). Similarly, the mean agreement rating for the shock-minimizing goal was significantly above the midpoint of the scale (Wilcoxon signed-rank test: *z* = 4.04, *P* < 0.001, effect size *r* = 0.58), while the mean agreement rating for the money-maximizing goal was significantly below the midpoint (Wilcoxon signed-rank test: *z* = −3.19, *P* = 0.0014, effect size *r* = 0.46).

When forced to choose one of the two goal statements to describe the goal of the peer, 44 out of 52 participants indicated that the antisocial peer's goal was better characterized by the money-maximizing statement (population proportion test against chance level, χ^2^ = 23.56, *P* < 0.001), while 36 out of 48 participants indicated that the prosocial peer's goal was better characterized by the shock-minimizing statement (χ^2^ = 11.02, *P* < 0.001).

## 3 Study 2

Study 1 confirmed our hypothesis that participants would accurately infer the goals of peers when observing how they decided to trade off profit for oneself against pain for a stranger. In Study 2, using computational modeling we investigated whether and how such peer observations would change the participants' own behaviors. Specifically, we tested four predictions. First, we predicted that after observing their peer's decisions, the participants' decisions would come to resemble those of the peers. Second, if inferring peers' goals influence participants' value computation in moral decision-making, we should observe that goal-consistent choice attributes (i.e., money for the participants observing the anti-social peer, pain for the participants observing the prosocial peer) should receive a higher weight in value computation following peer observation. Although participants in Study 2 did not explicitly report the goal of the peer, we assumed, based on the results of Study 1, that the information about the peer's goal should be available to them after the learning task. Third, peer influence might manifest as a shift in the initial choice bias, such that observing a prosocial peer would bias choices toward minimizing pain, while observing an antisocial peer would bias choices toward maximizing profit. Fourth, we predicted influence effects would scale with objective similarity between the participant and the peer. Lastly, we predicted that self-reported awareness of peer influence would track more closely with actual influence – as quantified by changes in the weights on pain and profit – for prosocial relative to antisocial influence. That is, people may be more accurate in reporting prosocial than antisocial influence effects.

### 3.1 Materials and methods

#### 3.1.1 Participants

For Study 2, healthy volunteers aged 18–40 years were recruited from the University of Oxford, Oxford Brookes University, and local residents of Oxford, the UK. The study was conducted at the Department of Experimental Psychology, University of Oxford, the UK, and was approved by the University of Oxford ethics committee (R50262/RE001). All participants gave written informed consent and were paid for their time. Each participant completed an hour-long battery of online personality questionnaires and demographic measures before attending a testing session that lasted approximately two hours (for details, please see Section 1.1 in **Supplementary materials)**. Participants with a history of neurological or neuropsychiatric disorders, pregnant women, and more than two years of study in psychology were excluded from participation. Participants who had previously participated in studies involving deception or electric shocks were also excluded due to concerns that prior experience with being deceived would influence belief in the outcomes of the current task, which did not involve deception.

Sample size was estimated based on a power analysis. According to a meta-analysis (Abrahamse & Steg, 2013), the effect size of social influence on moral behavior is small to medium (with a Hedges *g* = 0.35). Based on this information, we assumed a small effect size (f = 0.2) of interactions between group (prosocial vs. antisocial) and stage (baseline vs. post-influence) in the analysis of harm aversion parameters. Calculation based on G*Power (Faul, Erdfelder, Buchner, & Lang, 2009) indicated that thirty-four participants in each group were needed to detect a significant (α = 0.05) within-between interaction with a power (1 – β) of 0.9. We immediately checked participants' responses as the data came in (but we did not run any analysis for hypothesis testing at this stage), and excluded the participants whose responses met our exclusion criteria (see **3.1.4. Participant Exclusion**), until we reached our predetermined sample size. Ninety-one participants were recruited (see **3.1.2. Procedure** for details). After exclusion, we had 68 participants in the final dataset, 34 in the prosocial group (mean age 23.0 ± 4.7 years, 17 male, 17 female) and 34 in the antisocial group (mean age 22.5 ± 3.8 years, 17 male, 17 female).

#### 3.1.2 Procedure

The paradigm consisted of three stages (Fig. 1d): a baseline decision stage, a prediction stage, and a post-influence decision stage. In the baseline and post-influence decision stages, participants (in the role of the “decider”) completed a decision task as described in Study 1: in each stage, they made 48 private choices that involved trading money for themselves against moderately painful electric shocks for an anonymous stranger (the “receiver”), who was a real participant sitting in a neighboring testing room. We created two sets of 48 trials according to the same procedure as described in Study 1 (see Section 2.1.3. **Determination of trial sets: decision task**). For each participant, the same trial set was used for the baseline and post-influence decision stages, with the order of trials randomized. Participants were instructed that at the end of the experiment, one of their choices would be randomly selected and implemented. Thus, participants knew that their decisions could result in some amount of pain to the receiver, and that they could gain a monetary bonus for increasing the receiver's pain (the full instructions for Study 2 can be found in Section 1.2 in **Supplementary materials**).

In the prediction stage, participants were randomly assigned to predict either a prosocial peer (*N* = 34) or an antisocial peer (N = 34). The procedure was exactly the same as in Study 1, except that in Study 2 the participants were doing the task in a laboratory setting.

Finally, after the post-influence stage the participants answered post-task questions. There were four categories of questions: 1) morality and competence judgment of the peer, 2) participants' attitude toward the peer, 3) participants' perceived similarity with the peer and perceived changes in decision-making before and after the prediction stage, and 4) their emotional responses toward the peer's choices (for a complete list of these questions, please see Section 1.3 in **Supplementary material)**. Categories 1) and 2) were included as manipulation checks – we predicted that participants would judge the prosocial peer as more moral but not more competent than the antisocial peer, and would have more favorable attitudes toward the prosocial than the antisocial peers. These predictions were supported by the data (for details, please see Section 2.1 and **Fig. S1** in **Supplementary material**). Responses to category 3) were used to investigate whether perceived similarity and awareness of influence played a role in the objective changes in the participants' behaviors characterized by our computational models (please see sections **3.2.5** and **3.2.6** for details). Category 4) was included as exploratory measures (for details, please see **Fig. S1**).

#### 3.1.3 Computational modeling analysis of choice data

We modeled participants' behavior at baseline and post-influence using a utility model (Eq. 1) that quantifies the exchange rate between money and pain using: ∆V = (1- κ)∆m – κ∆s. The meaning of κ can be found in section **2.1.4. Determination of trial sets: prediction task**. Trial-by-trial ∆V was transformed into choice probabilities using a softmax function, which included an inverse temperature parameter β that served as an index of choice randomness (Eq. 4). A lapse rate parameter ε was also included in the softmax transformation to capture decision randomness resulting from factors other than value difference (such as inattention and erroneous responses; see Crockett et al., 2017):

(4) $$P\left(\mathrm{harm}\right)=\left(\frac{1}{1+e^{-\beta \Delta V}}\right)\left(1-2\varepsilon \right)+\varepsilon$$

To unpack the meaning of the ε parameter and Eq. 4, we rewrite Eq. 4 as follows:

(5) $$P\left(\mathrm{harm}\right)=\left(1-\varepsilon \right)P_{\mathrm{true}}+\varepsilon \left(1-P_{\mathrm{true}}\right),$$

where *P*_true_ = $\left(\frac{1}{1+e^{-\beta \Delta V}}\right)$ indicates the true probability of choosing the harmful option. The underlying assumption of Eq. 5’ is that the observed probability of harmful choice has two sources – the participant's true probability of choosing the harmful option (i.e., *P*_true_), and response error, where the participant intends to choose the helpful option but due to factors other than value difference (such as inattention and erroneous responses) mistakenly choose the harmful option (i.e., 1 – *P*_true_). The two sources of contribution to the observed *P*(harm) are weighted by the ε parameter. Larger ε indicates that more of the participant's choices are due to irrelevant factors and response errors.

For each participant, we estimated the free parameters for each of the two decision stages using nonlinear optimization implemented in Matlab (MathWorks) for maximum likelihood estimation. We fitted separate κ and β parameters for each decision stage. At the group level, non-parametric statistics were used to compare harm aversion in the first and second decision stages, as these parameters were not normally distributed (see **Table S3**).

#### 3.1.4 Participant exclusion

Among the ‘decider’ participants, thirteen were excluded from data analysis: data of two participants was missing due to technical errors; five participants explicitly expressed suspicion about whether the receiver would actually receive shocks; four did not find the electric shocks unpleasant; two mentioned that they adapted their decision strategy based on their suspicion about the receiver's gender. Another ten participants were excluded because their harm aversion (κ) in the baseline decision stage was more extreme than the harm aversion of the observed peer (i.e., they were less harm averse than the antisocial peer or more harm averse than the prosocial peer), which could result in an influence effect opposite to that which we had intended with the peer manipulation. It might be argued that such an exclusion criterion created an asymmetry in terms of their baseline moral preference between the two groups. To check if the behavioral changes we observed were robust, we applied a symmetric exclusion criterion to both groups, namely, excluding participants from both groups whose harm aversion in the baseline stage was either lower than 0.2 or higher than 0.8. The results still held under this exclusion criterion (for details, see **Section 2.4** in **Supplementary material**). The two groups did not differ with regard to demographic variables, manipulation check responses, or personality traits (see **Table S1**).

#### 3.1.5 Analysis of decision data: hierarchical drift-diffusion model (HDDM)

We used a Bayesian hierarchical drift diffusion model (HDDM) framework suited for estimating trial-by-trial parametric modulations on latent decision processes (Wiecki et al., 2013). A Bayesian estimation procedure was adopted to estimate the joint posterior distribution of model parameters based on observed decision data (i.e., reaction times and choices). This framework assumes that individual participants are random samples drawn from group-level distributions. Parameters of the preferred model were extracted from each participant and were subjected to individual difference analysis using Bayesian statistical tests (for details, please see **Section 3.1.6**. Analysis of individual differences in DDM parameters).

Following a standard procedure of HDDM model estimation (Wiecki et al., 2013), we used Markov chain Monte Carlo sampling methods for Bayesian approximation of the posterior distribution of parameters (generating 11,000 samples, discarding 1000 samples as burn-in). Here, harmful choices were coded as 1 and helpful choices were coded as 0. Reaction times (RT) longer than 20 s or shorter than 0.3 s were excluded (less than 2% of all trials). We inspected traces of model parameters, their autocorrelation and computed the R-hat (Gelman-Rubin) convergence statistics to ensure that the models had properly converged (Wiecki et al., 2013). Five chains were run, each with 5000 iterations and 200 burn-in samples. No R-hat statistics were larger than 1.1, indicating good convergence (Ulrichsen et al., 2020). Data from the baseline and the post-influence decision stages were entered the models simultaneously (for details of model structures, please see 3.2.3. **Money and pain drive value accumulation in moral decision-making**). Parameter distributions at both the group level and the individual-participant level are simultaneously estimated (Vandekerckhove, Tuerlinckx, & Lee, 2011). Deviance information criterion (DIC), suitable for hierarchical model comparison, was used as a measure of goodness-of-fit (Wiecki et al., 2013), with a difference of more than 10 considered significant (Herz et al., 2018). To evaluate if the preferred model can reproduce key patterns in the observation, we carried out posterior predictive checks, where we simulated data based on the parameters derived from the preferred model. This analysis showed that the preferred model satisfactorily reproduced the observed proportion of harmful decision and the means and the quantiles of RT for harmful and helpful decision (**Table S2,**Fig. 4). This indicates that the preferred model could reliably reconstruct the patterns in the observed data.

#### 3.1.6 Analysis of individual differences in DDM parameters

Bayesian general linear regression (the rstanarm package in R) was used to examine the relations between DDM parameters, including the changes in money- and pain-driven value accumulation (i.e., Δw_money_ and Δw_pain_) and changes in initial bias, on the one hand, and participants' objective and subjective similarity with the peer, and participants' self-reported perceived changes in behaviors across the two decision stages (i.e., awareness of influences) on the other hand. In these regression models, we included prediction accuracy as a covariate to control for the potential influences of variance in prediction accuracy. The rationale of adopting the Bayesian regression approach is that the DDM parameters were obtained via a Bayesian hierarchical estimation procedure (Markov chain Monte Carlo, MCMC) and it is inappropriate to apply frequentist statistics to MCMC estimates (Katahira, 2016; Boehm, Marsman, Matzke, & Wagenmakers, 2018).

### 3.2 Results

#### 3.2.1 Predictions and subjective impressions of peers reflect accurate moral inference

Prediction accuracy data indicated that participants successfully learned the prosocial and antisocial peers' moral preferences, with an overall accuracy of 87% by the final 10 trials of the prediction stage (antisocial group: M ± s.e.m. = 89 ± 2%; prosocial group: M ± s.e.m. = 85 ± 2%). This accuracy did not differ between groups (Mann-Whitney *U* test: *z* = −1.70, *P* = 0.089). Subjective impression ratings also indicated that participants distinguished between the prosocial and antisocial peers' moral character. Prior impressions did not differ between the two peers (antisocial group: M ± s.e.m. = 0.53 ± 0.01; prosocial group: M ± s.e.m. = 0.54 ± 0.03; Mann-Whitney *U* test: *z* = −0.54, *P* = 0.586). For the rest of the impression ratings, we used linear mixed-effects models to estimate how impressions differed between the two peers and how they evolved over time. Using a similar linear mixed-effect model described in Study 1, we found that, as in Study 1, impression of the prosocial peer was significantly more positive than the impression of the antisocial peer (*B* = 0.33 ± 0.04, CI = [0.25, 0.40], *t* = 8.05, *P* < 0.001). Additionally, we found that over time the difference between the impressions of the prosocial and the antisocial peers grew larger, as characterized by a significant group-by-time interaction on impression ratings (*B* = 0.012 ± 0.003, CI = [0.006, 0.018], *t* = 4.01, *P* < 0.001) (**Fig. S1a**). Post-task ratings confirmed that participants viewed the prosocial peer as more moral than the antisocial peer, and held more favorable attitudes toward the prosocial than the antisocial peer (**Fig. S1b-d**).

#### 3.2.2 Peer influence on moral decision-making

**Fig. 3:**
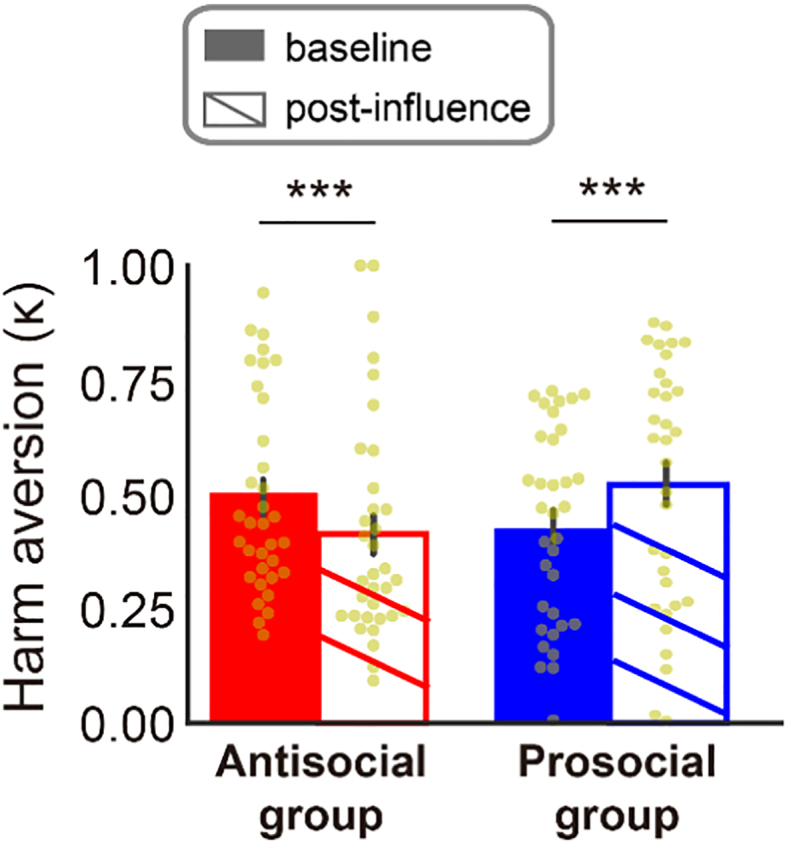
Harm aversion parameters in the baseline and post-influence decision stages. Relative to their harm aversion during the baseline decision stage, the harm aversion of the prosocial group increased (blue bars) while that of the antisocial group decreased (red bars) in the post-influence decision stage, indicating an alignment of preferences toward the peer. Error bars indicate s.e.m. ***: p < 0.001. Wilcoxon signed-rank test was used to determine significance. N = 34 for the Prosocial group (or peer), N = 34 for the Antisocial group (or peer). (For interpretation of the references to colour in this figure legend, the reader is referred to the web version of this article.)

The computational model fit participants' choices well, correctly predicting 89% of participants' choices in the baseline decision stage (95% confidence interval [87–90%]; mean pseudo-*r*^2^ = 0.630) and 90% in the post-influence decision stage (95% confidence interval [89–91%]; mean pseudo-*r*^2^ = 0.667). We hypothesized that participants who predicted the choices of the prosocial peer would become more harm averse, and that participants who predicted the choices of the antisocial peer would become less harm averse. Supporting our prediction, we observed a significant interaction between group (prosocial vs. antisocial peer) and stage (baseline vs. post-influence decision stage) on harm aversion (Friedman Rank Test, *F* (1, 66) = 41.23, *P* < 0.001). Neither the main effect of group (*F* (1, 66) < 0.001, *P* > 0.99) nor the main effect of stage (*F* (1, 66) = 0.84, *P* = 0.36) was significant (please also see Section 2.3 in **Supplementary materials)**. Specifically, we observed in the prosocial group a significant increase in harm aversion from the baseline to post-influence decision stage (κ_1_ = 0.44 ± 0.22, κ_2_ = 0.52 ± 0.27; Wilcoxon signed-rank test: *z* = 3.47, *P* < 0.001, effect size *r* = 0.60; Fig. 3, blue bars), whereas in the antisocial group we observed a significant decrease in harm aversion from the baseline to post-influence decision stage (κ_1_ = 0.50 ± 0.21, κ_2_ = 0.41 ± 0.24; Wilcoxon signed-rank test: *z* = 4.12, *P* < 0.001, effect size *r* = 0.71; Fig. 3, red bars). After predicting the choices of the peers, the prosocial group on average required 38% more money per shock to deliver extra shocks to the receiver, whereas the antisocial group on average required 31% less money per shock to deliver extra shocks to the receiver. These results provide evidence for peer influence on moral decision-making: observing the choices of a prosocial or antisocial peer shifted preferences to align with those of the peer. The absolute magnitude of changes in harm aversion did not significantly differ between groups (Mann-Whitney *U* test: *z* = 0.01, *P* = 0.99). In other words, the extent to which harm aversion increased after observing the prosocial peer was not significantly different from the extent to which harm aversion decreased after observing the antisocial peer. It is worth noting that baseline harm aversion did not significantly differ between groups (Mann-Whitney *U* test, *z* = −0.90, *P* = 0.371).

The inverse temperature parameter β did not differ significantly between the two decision stages, neither for the prosocial group (Wilcoxon signed-rank test: *z* = 1.27, *P* = 0.206) nor for the antisocial group (Wilcoxon signed-rank test: *z* = 1.22, *P* = 0.221**)**, indicating that observing peers' choices did not make participants' choices more or less stochastic.

#### 3.2.3 Money and pain drive value accumulation in moral decision-making

We next examined the extent to which value accumulation in moral decision-making was driven by relative money (∆m) and relative pain (∆s) between choice options. To this end, we combined the choice data of both groups from the baseline decision stage and modeled choices and RT with a multi-attribute drift-diffusion model (DDM) in which choice results from the noisy accumulation of a relative value signal that applies linear weights to money and pain (Wiecki et al., 2013). Four independent parameters describe the value accumulation process. The drift rate describes the speed of value accumulation favoring one option over the other and can be weighted by money, pain, or both; the decision threshold determines the boundary that the relative value signal favoring one choice option needs to reach for a decision to be executed; the initial bias quantifies the starting point of the value accumulation process before any information about choice attributes becomes available; and finally the non-decision time (NDT) summarizes aspects of RT that are not related to the value accumulation process, such as perception and motor response execution. We compared 6 models. In all of these models, threshold was modulated by the dummy variable indicating the baseline and post-learning stages. This was to account for the significant reduction in reaction times in the post-learning stage relative to the baseline stage (see section 2.5 and **Fig. S2** in **Supplementary material**). Moreover, in all of these models, the drift rate was weighted by relative money (w_money_) and relative pain (w_pain_) in a trial-by-trial manner, as an earlier study has shown that participants process both the relative money and relative pain information at the time of decision-making in this task (Crockett et al., 2017). In Model 1 through Model 4, the stage dummy variable modulated the weight on drift rate of relative money (w_money_), relative pain (w_pain_), both, or neither (Table 1). In all of these models, initial bias (z) was not modulated by trial-by-trial decision variables (Δs, Δm) or the stage dummy variable. Model comparison using Deviance Information Criterion (DIC) indicated that among these 4 models, the model where the stage dummy variable modulated both w_money_ and w_pain_ was preferred (Table 1). On the basis of this model, we further demonstrated that allowing initial bias to vary across stages further improved model fitting (Model 5). Finally, as a comparison, we added the stage-dependent initial bias term to Model 1 to test whether including this term alone (i.e., Model 6) could improve model fitting over and above the preferred model without this term. However, this was not the case: Model 6 was outperformed by all the models except Model 1.

**Table 1:** Drift-diffusion model structure and comparison.

Model	Parameters	DIC (prosocial)	DIC (antisocial)
Model 1	v ~ 1 + w_money_Δm + w_pain_Δs a ~ 1 + stage z ~ 1	12,676	13,529
Model 2	v ~ 1 + w_money_Δm * stage + w_pain_Δs a ~ 1 + stage z ~ 1	12,507	13,334
Model 3	v ~ 1 + w_money_Δm + w_pain_Δs * stage a ~ 1 + stage z ~ 1	12,483	13,376
Model 4	v ~ 1 + w_money_Δm * stage + w_pain_Δs * stage a ~ 1 + stage z ~ 1	12,448	13,311
**Model 5**	v ~ 1 + w_money_Δm * stage + w_pain_Δs * stage a ~ 1 + stage z ~ 1 + stage	**12,425**	**13,290**
Model 6	v ~ 1 + w_money_Δm + w_pain_Δs a ~ 1 + stage z ~ 1 + stage	12,533	13,395

Notes: w_money_ and w_pain_ represent modulation (or weight) of the relative money and relative pain on drift rate. “1” stands for a participant-specific constant (or intercept). a = decision threshold, z = initial bias, and v = drift rate. In all of these models, non-decision time (NDT) was not modulated by trial-by-trial parameters (Δm, Δs) or decision stage. DIC = deviance information criterion.

**Fig. 4:**
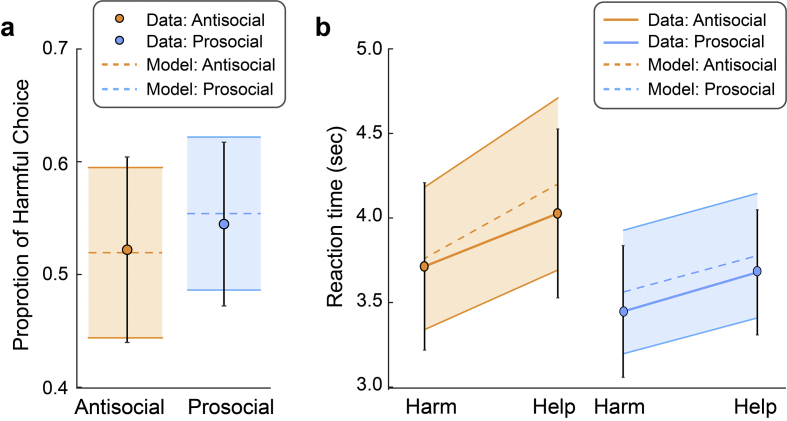
Accuracy of the DDM models in predicting choice and RT data. (a-b) Observed (dots and solid lines) and simulated (dotted lines) choice proportion (a) and reaction times (b) for the antisocial and prosocial datasets. Shaded areas are the 95% confidence intervals of the simulated data (see also Table S2). Matlab codes for plotting panels (a) and (b) were adapted based on Rollwage, Loosen, Hauser, Moran, Dolan, & Fleming. (2020). Confidence drives a neural confirmation bias. Nature Communications, 11(1), 2634.

To evaluate the goodness-of-fit of the favored model (Model 5), we simulated choices and RT based on the model parameters estimated from Model 5. Both the observed choice proportion and RT were within the 95% confidence interval of the model predicted values, for both the antisocial and the prosocial groups (Fig. 4**; Table S2**).

#### 3.2.4 Observing peer's decisions modulates choice bias and value accumulation

**Fig. 5:**
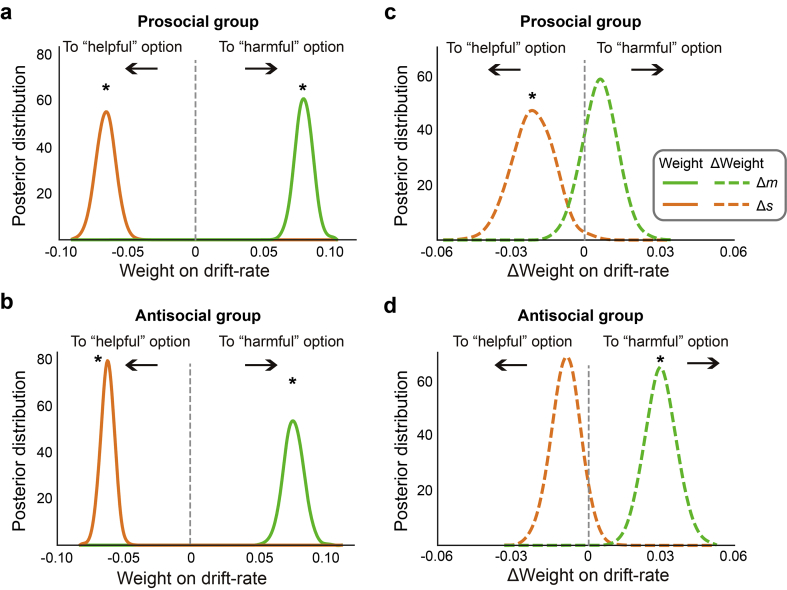
Prosocial and antisocial influence on money- and pain-driven value accumulation. (a-b) Posterior probability distributions of the main effect of relative money and relative pain on drift rate (w_money_ and w_pain_) were significant both for the prosocial and for the antisocial group. (c-d) Predicting the prosocial peer selectively enhanced the weight of relative pain on drift rate (c) whereas predicting the antisocial peer selectively enhanced the weight of relative money on drift rate. *: posterior probability > 95%.

Based on the preferred models, trial-by-trial decision variables (Δs, Δm) had significant impact on the drift rate for both the prosocial and antisocial groups – relative money increased drift rate toward the harmful option, whereas relative pain increased the drift rate toward the helpful option (all posterior probabilities >99%; Fig. 5a and b). Crucially, for the prosocial group, the weight on relative pain but not on relative money was strengthened after learning (the probability of Δw_pain_ being negative is 99%, the probability of Δw_money_ being positive is 79%; Fig. 5c); in contrast, for the antisocial group, the weight on relative money but not on relative pain was strengthened after learning (the probability of Δw_pain_ being negative is 88%, the probability of Δw_money_ being positive is higher than 99%; Fig. 5d). The interaction effects shown in Fig. 5c and Fig. 5d can be seen in the majority of individual participants (**Fig. S3**).

**Fig. 6:**
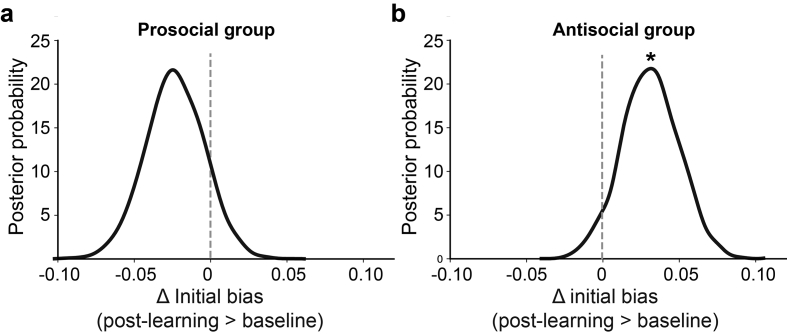
Learning-induced changes in initial bias. (a) Predicting a prosocial peer tended to shift the initial bias toward the helpful option (although not statistically significant, poster probability = 90%). (b) Predicting an antisocial peer shifted the initial bias toward the harmful option (poster probability = 96%). *: posterior probability >95%.

Relative to the baseline stage, participants' decision threshold significantly decreased, regardless of which peer they predicted (posterior probabilities >99%), and there was no significant difference in the magnitude of reduction between the two groups (probability = 70%). Initial bias significantly increased for the antisocial group (Fig. 6b), suggesting that after predicting the antisocial peer (probability = 96%), the participants were more inclined to choose the harmful option regardless of the decision variables (Δs, Δm). An opposite trend was observed for the prosocial group, indicating the after predicting the prosocial peer the participants were more inclined to the helpful option (Fig. 6a), although the shift was not statistically significant (probability = 90%). Because the initial bias changed in opposite directions, the relative shift of initial bias between the two groups were significant (probability = 98%).

#### 3.2.5 Objective and subjective similarity with the peer enhances influence effects on drift weights and initial bias

In line with prior work suggesting people are more likely to adopt the goals and preferences of similar than dissimilar others (Izuma & Adolphs, 2013; Shang, Reed, & Croson, 2008), we hypothesized that participants would be more likely to adjust their preferences to conform to peers whose preferences were more similar to theirs. We considered two metrics of similarity: self-reported perceptions of similarity with the peer (the aspect of similarity that has been examined in past social influence research), and the “objective similarity” in actual preferences. Here, objective similarity with the peer was defined as reversed distance (i.e., 1 – distance) between the peer's harm aversion and the participant's baseline harm aversion (i.e., 1 – (κ_1_–0.2) for the antisocial group, 1 – (0.8 – κ_1_) for the prosocial group). The objective similarity defined this way did not differ between the groups (Mann-Whitney *U* test: *z* = −0.97, *p* = 0.333). We predicted that for the prosocial group, objective similarity at baseline should be predictive of the changes in the pain-driven value accumulation but not the changes in the money-driven value accumulation. In contrast, for the antisocial group, objective similarity at baseline should be predictive of the changes in the money-driven value accumulation but not the changes in the pain-driven value accumulation.

**Fig. 7:**
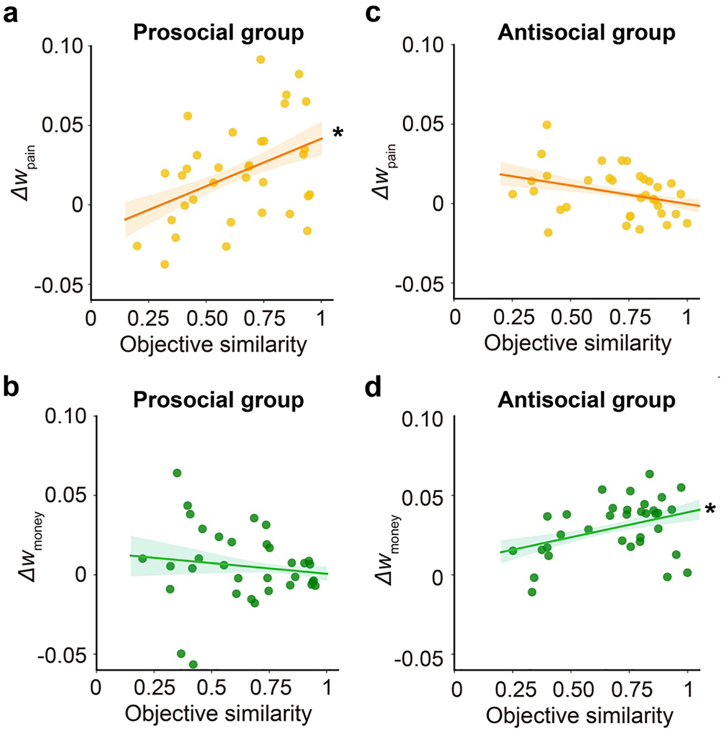
Objective similarity with peer modulates influence effects on value accumulation. (a-b) For the prosocial group, objective similarity with the peer was positively correlated with changes in pain-driven value accumulation (Δw_pain_), but not with changes in money-driven value accumulation (Δw_money_). (c-d) For antisocial group, objective similarity with the peer was positively correlated with changes in money-driven value accumulation (Δw_money_) but not changes in pain-driven value accumulation (Δw_pain_). * indicates a significant effect (i.e., credible interval excludes 0).

To test this, in two separate regression models we regressed the changes in pain-driven and money-driven value accumulation (i.e., Δw_pain_ and Δw_money_) against their objective similarity with the peer, the peer they predicted (prosocial vs. antisocial), and the interaction between the two (see **3.1.6. Individual differences analysis on DDM parameters**). For both regression models, the interaction between peer and objective similarity was significant (*B* = 0.080 ± 0.026, credible interval = [0.038, 0.122] for Δw_pain_, **Fig. S4a**; *B* = −0.046 ± 0.022, credible = [−0.081, −0.010] for Δw_money_, **Fig. S4d**), indicating that the relationship between objective similarity and changes in pain-driven and money-driven value accumulation varied as a function of the peer predicted. Specifically, the association between objective similarity and Δw_pain_ was only significant for the prosocial group (*B* = 0.060 ± 0.019, credible interval = [0.031, 0.090]; Fig. 7a**, Fig. S4b**), but not for the antisocial group (*B* = −0.022 ± 0.019, credible interval = [−0.053, 0.009]; Fig. 7c**, Fig. S4c**). Conversely, Δw_money_ was correlated with objective similarity in the antisocial group (*B* = 0.032 ± 0.017, credible interval = [0.004, 0.059]; Fig. 7d**, Fig. S4f**), but not the prosocial group (*B* = −0.015 ± 0.016, credible interval = [−0.041, 0.011]; Fig. 7b**, Fig. S4e**). In other words, prosocial peers were more effective at increasing pain-driven value accumulation in more harm-averse participants, while antisocial peers were more effective at increasing money-driven value accumulation in less harm-averse participants. This pattern was replicated using two alternative analytic strategies (for details, please see Section 2.6 and **Fig. S5** in the **Supplementary material**).

**Fig. 8:**
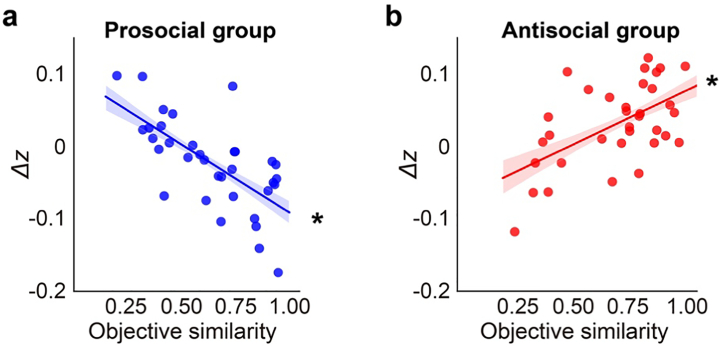
Objective similarity with peer modulates influence effects on initial bias. (a) Objective similarity with the prosocial peer was negatively correlated with changes in initial bias (post-learning – baseline), indicating that the participants who were more objectively similar to the prosocial peer became more biased toward the helpful option after the learning stage. (b) Objective similarity with the antisocial peer was positively correlated with changes in initial bias, indicating that the participants who were more objectively similar to the antisocial peer became more biased toward the harmful option after the learning stage. * indicates a significant effect (i.e., credible interval excludes 0).

Next, we examined whether objective similarity had an impact on the shift of initial bias. We regressed the changes in initial bias against the participants' objective similarity with the peer, the peer they predicted (prosocial vs. antisocial), and the interaction between the two. Prediction accuracy in the learning stage was included as a covariate. Both the main effect of objective similarity (*B* = 0.145 ± 0.039, credible interval = [0.079, 0.205]) and the interaction between peer and objective similarity (*B* = −0.331 ± 0.054, credible interval = [−0.420, −0.249]) were significant. Specifically, the association between objective similarity and changes in initial bias was significant both for the prosocial group (*B* = −0.186 ± 0.038, credible interval = [−0.246, −0.125]; Fig. 8a), and for the antisocial group (*B* = 0.149 ± 0.040, credible interval = [0.081, 0.207]; Fig. 8b).

We tested whether similar effects of similarity would be observed when considering participants' subjective perceptions of similarity reported in the post-task debriefing questionnaire (“How similar do you feel to [peer's initial]?”; continuous scale ranging from 0 = not at all similar to 100 = very similar). We found that subjective similarity (re-scaled to 0–1) with the prosocial peer, but not the antisocial peer, was positively correlated with changes in pain-driven value accumulation w_pain_ (prosocial peer: *B* = 0.050 ± 0.016, credible interval = [0.025, 0.074]; antisocial peer: *B* = −0.016 ± 0.017, credible interval = [−0.042, 0.011]), and negatively with changes in money-driven value accumulation w_money_ (prosocial peer: *B* = −0.035 ± 0.013, credible interval = [−0.056, −0.014]; antisocial peer: *B* = 0.008 ± 0.014, credible interval = [−0.015, 0.030]). The difference between prosocial and antisocial peer was evidenced by significant interactions between subjective similarity and peer (for Δw_pain_: *B* = 0.065 ± 0.023, credible interval = [0.031, 0.102]; for Δw_money_: *B* = −0.043 ± 0.019, credible interval = [−0.075, −0.013]). These relations held even when the objective similarity was included as a covariate in the regression (please see Section 2.7 in the **Supplementary materials** for details).

Finally, we examined whether subjective similarity was associated with the shift of initial bias. Like we did with objective similarity, we regressed the changes in initial bias against the participants' subjective similarity with the peer, the peer they predicted (prosocial vs. antisocial), and the interaction between the two. Prediction accuracy in the learning stage and objective similarity with the peer were included as covariates. Both the main effect of subjective similarity (*B* = 0.080 ± 0.040, credible interval = [0.019, 0.146]) and the interaction between peer and subjective similarity (*B* = −0.218 ± 0.052, credible interval = [−0.304, −0.140]) were significant. Specifically, the association between subjective similarity and changes in initial bias was significant both for the prosocial group (*B* = −0.130 ± 0.035, credible interval = [−0.203, −0.070]), and for the antisocial group (*B* = 0.089 ± 0.037, credible interval = [0.018, 0.148]).

#### 3.2.6 Asymmetric awareness of prosocial and antisocial influence

Were participants aware of the effects of peer influence on their moral decisions? At the end of the study, we asked the participants “To what extent did you choose differently after you observed [initials of the peer] compared with before?” on a 0 (not at all) – 100 (very much) continuous analog scale, as a measure of their perceived shift of moral preference. The two groups did not differ in the extent of the overall perceived shift (Mann-Whitney *U* test: *z* = −0.63, *P* = 0.508).

**Fig. 9:**
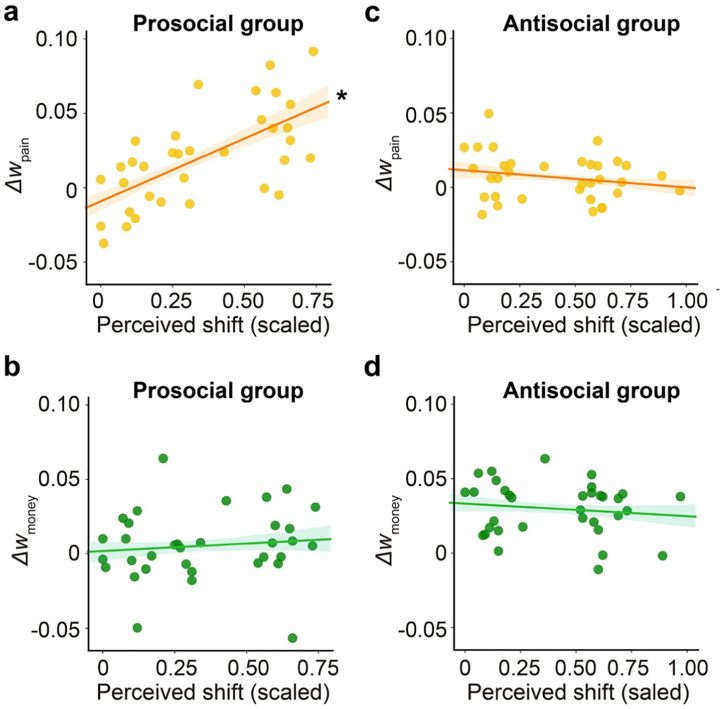
Awareness of prosocial, but not antisocial, influence tracks the influence effect. (a-b) For the prosocial group, perceived shift after the prediction stage was positively correlated with changes in pain-driven value accumulation (Δw_pain_), but not with changes in money-driven value accumulation (Δw_money_). (c-d) For the antisocial group, perceived shift was neither correlated with the changes in money-driven value accumulation, nor with the changes in pain-driven value accumulation. * indicates a significant effect (i.e., credible interval excludes 0).

To examine whether the awareness of influence was predictive of the influence effects that actually manifested in value accumulation, in two separate regression models we regressed changes in pain-driven and money-driven value accumulation (i.e., Δw_pain_ and Δw_money_) against the participants' perceived shift (re-scaled to 0–1), the peer they predicted (prosocial vs. antisocial), and the interaction between the two. As we did in the regressions regarding objective similarity, prediction accuracy in the learning stage was included as a covariate. For the regression with the changes in pain-driven value accumulation (Δw_pain_), we found a significant interaction between perceived shift and peer observation condition (*B* = 0.099 ± 0.020, credible interval = [0.067, 0.131]). Specifically, perceived shift was positively correlated with Δw_pain_ for the prosocial group (*B* = 0.084 ± 0.015, credible interval = [0.060, 0.106]; Fig. 9a), but not with the antisocial group (*B* = −0.014 ± 0.014, credible interval = [−0.035, 0.008]; Fig. 9c). The main effect of perceived shift was not significant (*B* = −0.014 ± 0.014, credible interval = [−0.036, 0.007]). For the regression with the changes in money-driven value accumulation (Δw_money_) (Fig. 9b**,**Fig. 9d), neither the main effect of perceived shift (*B* = −0.013 ± 0.014, credible interval = [−0.035, 0.008]), nor the interaction between perceived shift and the peer observation condition were significant (*B* = 0.025 ± 0.021, credible interval = [−0.008, 0.058]). Taken together, these findings suggest an asymmetry in the awareness of prosocial and antisocial moral influence: awareness of prosocial, but not antisocial influence, was informative of the magnitude of influence effect on the value accumulation processes.

To examine whether the awareness of influence was predictive of the influence effects manifested in initial bias, we regressed changes in initial bias against the participants' perceived shift (re-scaled to 0–1), the peer they predicted (prosocial vs. antisocial), and the interaction between the two. Prediction accuracy in the learning stage was included as a covariate. Neither the main effect of the perceived shift (*B* = 0.016 ± 0.040, credible interval = [−0.051, 0.077]) nor the interaction (*B* = −0.070 ± 0.059, credible interval = [−0.159, 0.027]) was significant, suggesting that although predicting a peer shifted participants' initial bias in a peer-specific manner, such shift does not contribute to participants' awareness of behavioral changes.

## 4 General discussion

In this study, we provide evidence that is consistent with our hypothesis that peer influence alters value computation in moral decision-making by causing people to prioritize choice attributes that are consistent with the peers' goals. Specifically, in Study 1 we showed that when observing prosocial peers making moral decisions, participants correctly inferred their peer prioritized minimizing others' pain. Meanwhile, participants correctly inferred that antisocial peers prioritized maximizing their own profits. In Study 2, we demonstrated that observing the choices of prosocial peers increased pain-driven value accumulation and biases toward the helpful option, while observing the choices of an antisocial peer increased money-driven value accumulation and biases toward the harmful option. These distinct effects of prosocial and antisocial influence occurred independently from effects of peer influence on choice bias, and were stronger in participants whose initial moral preferences were more similar to those of the observed peer. While participants reported low-to-moderate awareness of these influence effects, reported awareness tracked more closely with actual prosocial influence than antisocial influence. It should be noted that because goal inferences and moral value computations were measured in separate studies, strictly speaking we cannot draw conclusions regarding the causal relationships between these two processes. Future work should test this directly in the same study to assess whether there is a causal link between people's inferences of other's goals and behavioral change.

Our findings demonstrate how computational approaches can reveal novel insights into the processes that guide social decision-making (Crockett, 2016; Konovalov, Hu, & Ruff, 2018). By directly comparing prosocial and antisocial influence within the same computational framework, we were able to uncover a common computational process that characterizes moral influence. Consistent with work on goal contagion (Aarts et al., 2004; Dijksterhuis & Aarts, 2010), our findings suggest people quickly infer the goals of peers from observing their behavior. Moreover, the weight of goal-consistent choice attributes on value accumulation increased in observers' own decision-making following peer observation, independently from peer observation effects on initial choice bias. Past work on value-based decision-making shows that value accumulation is sensitive to attention. Specifically, attended attributes drive value accumulation more strongly than non-attended attributes (Krajbich, 2018). This suggests possible interventions to promote prosocial behavior or discourage antisocial behavior. For instance, if antisocial influence works via amplifying the impact of selfish benefits on value accumulation, it may be possible to dampen antisocial influence by drawing people's attention toward the harmful impact on others, which could counteract that value accumulation process and perhaps even reverse it (Kappes et al., 2018; Krajbich & Rangel, 2011). Meanwhile, highlighting the personal benefits of moral behavior may do little to enhance prosocial influence if this process operates primarily by increasing the impact of others' welfare on value accumulation.

Our findings suggest that peer influence on moral behavior can, at least in some cases, alter the value of moral behavior itself, independently from merely inducing superficial compliance with or imitation of others' actions. Our participants were explicitly instructed that their choices were unobserved and that they would not interact with either the peer or with the receiver who would receive electric shocks resulting from their choices. This aspect of our design minimized the possibility that reputational concerns induced participants to modify their behavior. Furthermore, computational analysis showed that learning the peer's moral preference altered subsequent value accumulation during moral decision-making, independently from impacting initial choice bias, which may reflect imitative compliance (Chartrand & Bargh, 1999; Chartrand & Lakin, 2013; Heyes, 2011). Our findings are consistent with previous work demonstrating that observing others' choices can modulate underlying valuation processes in risky decisions (Chung, Christopoulos, King-Casas, Ball, & Chiu, 2015), inter-temporal choice (Garvert, Moutoussis, Kurth-Nelson, Behrens, & Dolan, 2015), purchasing behaviors (Izuma & Adolphs, 2013), and aesthetic judgments (Zaki, Schirmer, & Mitchell, 2011). Privately adopting the moral preferences of others may be an adaptive strategy for preserving one's reputation when it cannot be certain whether decisions are observed by others (Bear, Kagan, & Rand, 2017).

Previous studies using the DDM to investigate the cognitive mechanisms underlying conformity primarily investigated conformity to a group's consensus (Germar et al., 2014; Son et al., 2019; Tump et al., 2020) rather than compliance or emulation, which may rely on different cognitive mechanisms (Cialdini & Goldstein, 2004; Kristjánsson, 2006). Furthermore, similarity between oneself and one's role model may have dissociable impacts on the different cognitive components underlying moral emulation, which has not been investigated by previous work. Our study fills this gap by combining the DDM framework with harm-based moral decision-making and learning tasks and demonstrated that observing the decisions of a peer makes goal-consistent decision attributes contribute more strongly to the observer's subsequent valuation. Because value accumulation has previously been linked with attention (Krajbich et al., 2010; Smith & Krajbich, 2019), we speculate that inferring the peer's goal directs the observer's attention to goal-consistent choice attributes, biasing value accumulation. Future research combining this paradigm with manipulations of perceptual attention or eye-tracking is needed to further discern the role of attention in peer influence.

Consistent with past findings, we found that prosocial and antisocial influence effects were amplified in participants whose preferences were more similar to the peer (Gino et al., 2009; Izuma & Adolphs, 2013; Platow, Mills, & Morrison, 2000; Shang et al., 2008). Most past research on similarity and influence has focused on subjective perceptions of similarity between observer and influencer, or on similarity along dimensions that are unrelated to the behavior being influenced. In contrast, our methods allowed us to measure objective similarity between observers and peers on the actual behavior being influenced. Our observation that objective similarity amplified influence effects, both in terms of moral valuation and of imitation, suggests an important potential limitation of social influence, particularly when it comes to inspiring moral change: peers who are objectively very different from oneself may ultimately be less effective influencers, even when the relevance or attainability of the exemplar's achievements is emphasized (Han, Kim, Jeong, & Cohen, 2017). As a technical note, it is worth mentioning that we replicated this finding using three distinct analysis strategies. We believe this to be a useful contribution to the literature in general because it illustrates how different analytic strategies can be used to examine individual differences in parameters estimated with a hierarchical Bayesian approach, which has been more and more widely adopted in social and cognitive psychology (e.g., Etz & Vandekerckhove, 2018).

Although our study directly investigated the effect of objective similarity in a morally relevant dimension on moral influence, this paradigm has the potential to be adapted to answer questions of how morally irrelevant similarities, such as social distance, group membership, and even physical appearance, modulate the effectiveness of prosocial and antisocial peers' influence. Previous research has shown that group membership plays a key role in the magnitude and direction of conformity. For example, while conforming to one's ingroup regarding a product preference caused lower psychological resistance relative to disagreeing, conforming to the preference of a morally opposed group caused more psychological resistance relative to disagreeing (Stein, 2017). From a different perspective, Gino and Galinsky (2012) have shown that psychological closeness with a person engaging in selfish or dishonest behavior leads people to behave more selfishly themselves, through a mechanism the authors termed ‘vicarious justification’. These studies did not examine whether group membership or closeness have asymmetric effects on prosocial versus antisocial influence, or whether similarity in one dimension (e.g., group membership) interacts with similarity in another dimension (e.g., moral preference) to determine the effect of moral influence.

Manipulating similarity in a dimension orthogonal to moral preference itself may help to delineate the causality between perceived similarity with the peers and the influence effect. Our finding that perceived similarity was associated with prosocial but not antisocial influence should be interpreted with caution. It is possible that participants perceived the peer as more similar to themselves and more relatable, and as a result modified their behaviors more to align with the peer. However, because the perceived similarity was measured after the behavioral task, it is also possible that the participants had an awareness of their behavioral changes and used that as a reference when evaluating perceived similarity. Future studies combining social similarity manipulations (e.g., group membership, closeness) with manipulation of peer preferences will be able to systematically address these questions.

Our computational approach also allowed us to measure the extent to which people are accurate in their reporting of the extent to which they have been influenced by their peers. While some past work suggests peer influence is under-detected (Bargh et al., 2001; Nolan et al., 2008), there is also evidence that people are able to accurately report components of their decision processes (Fleming & Dolan, 2010; Desender, Boldt, Verguts, & Donner, 2019). Here, we find that the magnitude of self-reported awareness of influence did not differ between positive and negative influence. However, self-reported awareness of influence tracked with actual influence only when that influence was positive. This finding comports with previous studies showing that people have less vivid and accurate memories of their unethical behaviors, relative to ethical behaviors (Carlson, Maréchal, Oud, Fehr, & Crockett, 2020; Kouchaki & Gino, 2016). People may be less willing or less able to accurately detect antisocial influence than prosocial influence because they are strongly motivated to preserve a moral self-image (Gino, Norton, & Weber, 2016; Mazar et al., 2008). This asymmetry in the accuracy of awareness of influence suggests antisocial influence is pernicious not just because it amplifies selfish preferences, but also because people may be unwilling or unable to detect its occurrence.

Although our study samples were balanced on gender, our study included predominantly White participants from the UK. Research on social influence effects across cultures suggests that many of our key findings may generalize across diverse samples, but also suggests there may be some important differences. For example, parental influence on substance use (e.g., alcohol, cigarettes) has been found both in European-American and in Asian-American adolescents (Au & Donaldson, 2000). Nevertheless, culture does make an impact on the susceptibly to social influence. For instance, Kongsompong, Green, and Patterson (2009) found that collectivism, which is more pronounced in Eastern than in Western cultures, is positively correlated with the effect size of social influence on purchasing decisions. The impact of culture on social influence seems to be stronger when the influence comes from an in-group member (e.g., parents) than from an out-group member (e.g., salesperson) (DeMotta, Kongsompong, & Sen, 2013). Altogether, this research suggests that our findings are likely to generalize to non-Western populations, and that the effects could be stronger in cultures where collectivism dominates.

Another limitation of our study is that we do not have a non-social control condition (e.g., Chierchia et al., 2020), and therefore cannot completely disentangle the effects of social influence and time (or mere repetition effect). Nevertheless, it is worth noting that any effects of task repetition would be matched between the prosocial and antisocial conditions, but the effects we observed in this study are mostly condition-specific, and prosocial and antisocial influence impacted moral decision-making in opposite directions and modulated distinct components of value accumulation. This would not be the case if the effects were solely driven by task repetition, which was matched between prosocial and antisocial conditions.

To conclude, our study provides a computational account of how observing a peer's behavior alters the valuation processes that underlie moral decision-making. By characterizing peer influence with a value accumulation process and linking its effects to observer's own preferences, we highlight a role for preference similarity in mediating influence effects, which may help improve the design of interventions for promoting prosocial behavior and preventing antisocial behavior.

## Author note

Data are available on the Open Science Framework (OSF) at https://osf.io/kwczf

The computational modeling code used to generate the analyses in the present study is available at the OSF address above. The design and analysis plan for Study 1 were preregistered at (https://aspredicted.org/8xw3q.pdf).

## Funding

This work was supported by the 10.13039/100000925John Templeton Foundation Beacons Project and the 10.13039/501100000691Academy of Medical Sciences (SBF001\1008). H.Y. was supported by The 10.13039/501100000288Royal Society Newton International Fellowship (NF160700). J.Z.S. was supported by a Clarendon and 10.13039/100010269Wellcome Trust Society and Ethics award (104980/Z/14/Z). The funders had no role in study design, data collection and analysis, decision to publish or preparation of the manuscript.

## Appendix A Supplementary data

Supplementary material Image 1
