# Supplementary material for: How peer influence shapes value computation in moral decision-making
Source: Cognition. 2021 Jun;211:104641. doi: 10.1016/j.cognition.2021.104641 (PMC8085736; doi:10.1016/j.cognition.2021.104641)
Supplement: Supplementary file 1 — Supplementary material [file mmc1.docx]

**Supplementary material for**

**How peer influence shapes value computation in moral decision-making**

*Hongbo Yu^1^, Jenifer Z. Siegel^1^, John A. Clithero^2^, Molly J. Crockett^1^*

^1^ Department of Psychology, Yale University, New Haven, Connecticut, USA

^2^ Lundquist College of Business, University of Oregon, Eugene, Oregon, USA

**This file includes:**

Supplementary Methods of Study 2

Supplementary Results of Study 2

Supplementary Tables 1 – 4

Supplementary Figure 1 – 5

1. **Supplementary** **Methods of Study 2**
   1. **Online personality questionnaires and demographic measures.**

Participants completed a battery of online personality questionnaires (Psychopathy, Social Desirability, Trait Anxiety Scale, Emotion Regulation, Interpersonal Reactivity Index, Moral Identity Scale, Oxford Utilitarianism Scale) and demographic measures (age, sex, ethnicity, years of education, income and religious belief) before attending the laboratory session.

- 1. **Full instruction for the moral influence paradigm.**

We presented the instruction to the participants in PowerPoint slides. We first showed the participants instruction for the baseline decision stage:

*You have been randomly assigned to the role of Decider. The other participant in this session has been randomly assigned to the role of Receiver.*

*As the Decider, you will make a series of decisions. Each decision involves choosing between a smaller amount of money plus a smaller number of shocks, or a larger amount of money plus a larger number of shocks.*

*As the Decider, you always receive the money, and the Receiver always receives the shocks.*

*The shock intensity level is always set to level 8 – just below the “intolerable” pain level. We will use the voltage setting that corresponds to the Receiver’s level 8 rating. This accounts for individual differences in pain tolerance.*

*Next, let’s see what the computer screens will look like:*

*You will first see two options:*

**
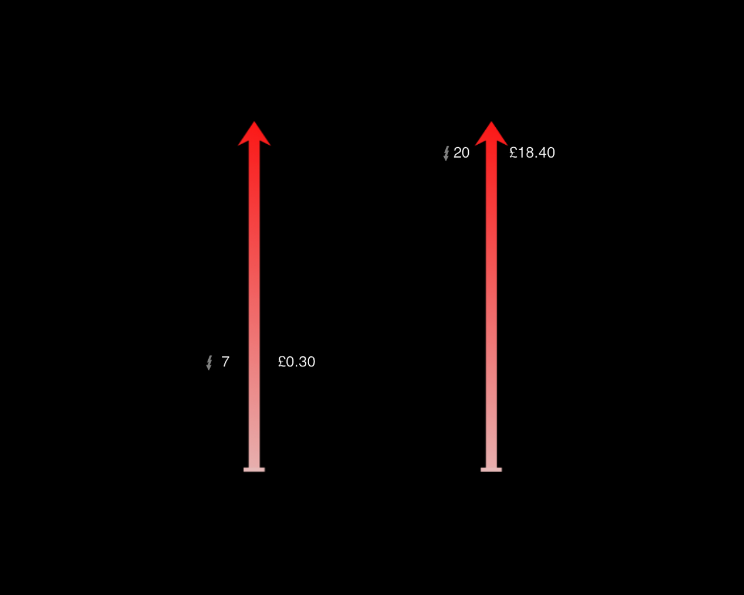
**

*You will then use the left or right mouse button to choose the option you prefer.*

*The chosen option will become highlighted:*

**
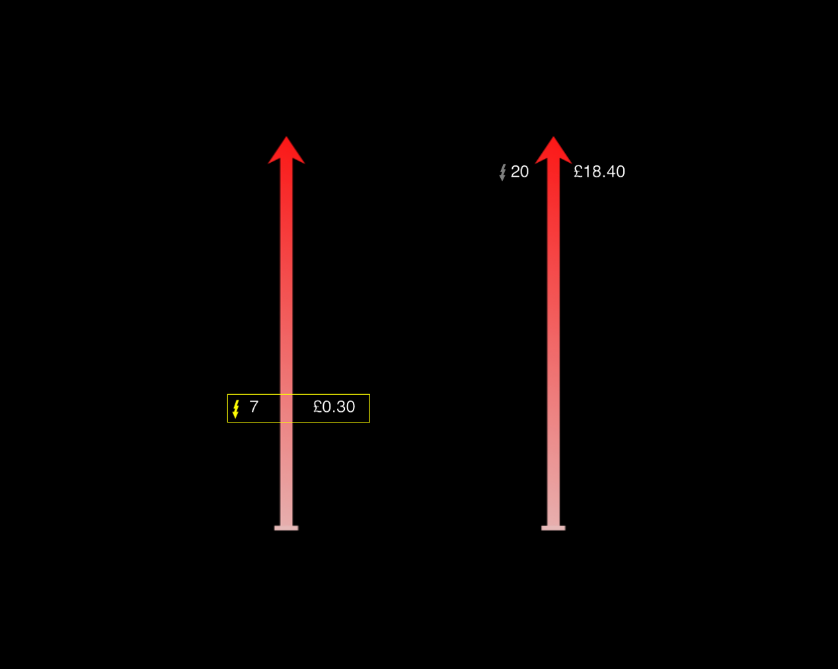
**

*Throughout the study you will make a series of decisions like these (approximately 50). However, no shocks will be delivered during the decision-making task.*

*Instead, at the end of the task, one trial will be randomly selected, and your decision from that trial will be actually implemented. You will receive the money, and the Receiver will receive the shocks.*

*Note that only one decision will count “for real”, so you do not need to worry about spreading the money or the shocks across the different trials. Since you will not know which trial will count, you should treat each decision as if it were the only one.*

*The Decision Task will last approximately 15 minutes.*

*When you have finished the Decision Task, or if you have any questions at any time, please notify the experimenter using the buzzer.*

Then when the participants finished the baseline decision stage, an experimenter would enter the cubicle to show the participants instructions for the prediction stage and the post-influence decision stage:

*Next, we would like you to complete a Prediction Task.*

*In this task, you will predict the decisions of another Decider, “MJ” who has previously faced choices similar to the ones you have just encountered (approximately 40).*

*One each trial, you will first see MJ’s options. Next, you will predict MJ’s choice. Finally, we will show you MJ’s choice and give you feedback about your accuracy. Try to be as accurate as possible in your predictions. You can earn additional money based on making accurate predictions.*

*The computer screens for the Prediction Task will look similar to the screens for the Decision Task, but the background will be blue instead of black. Also, after you have clicked on an option, you will receive feedback indicating whether your prediction was correct or not.*

*Let’s see what the computer screens will look like:*

*You will first see MJ’s options:*

*
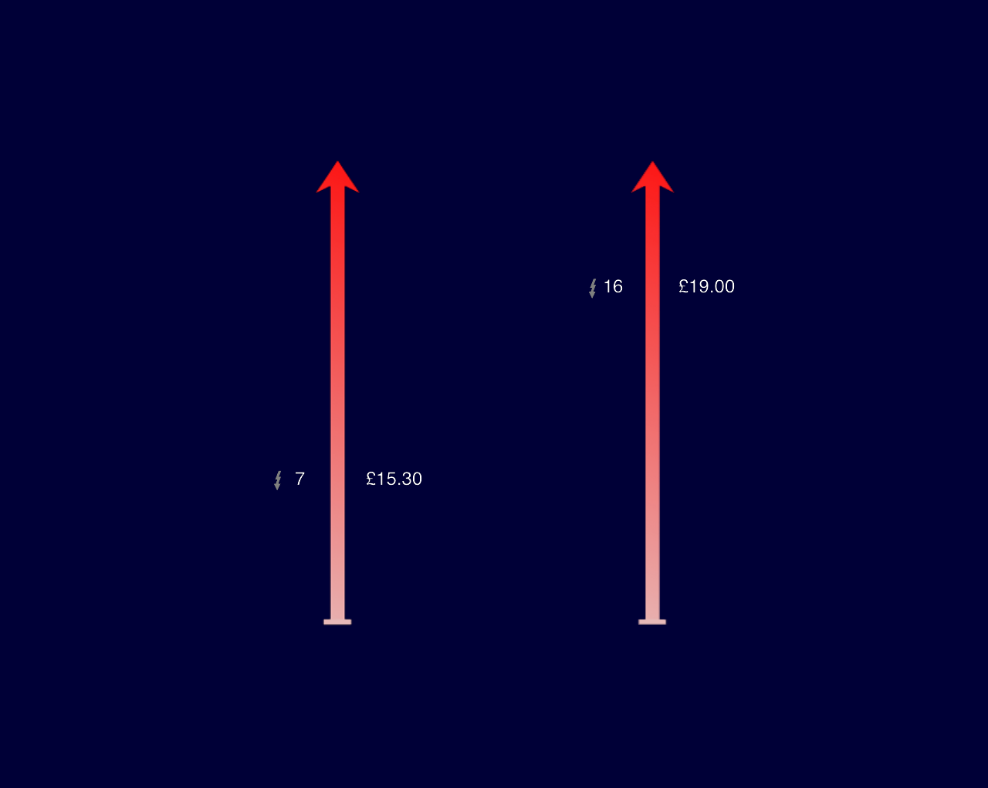
*

*Using the left or right mouse key, you will select the option you think MJ chose:*

*
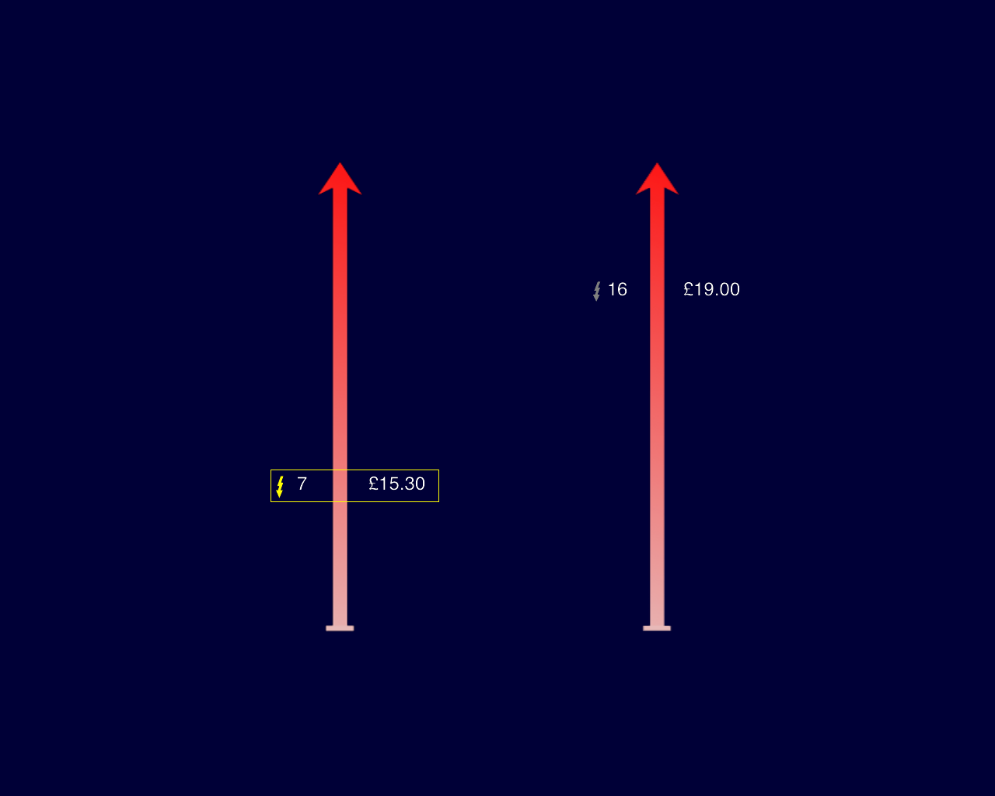
*

*You will then receive feedback about the accuracy of your prediction. If you are correct, this is what the screen will look like:*

*
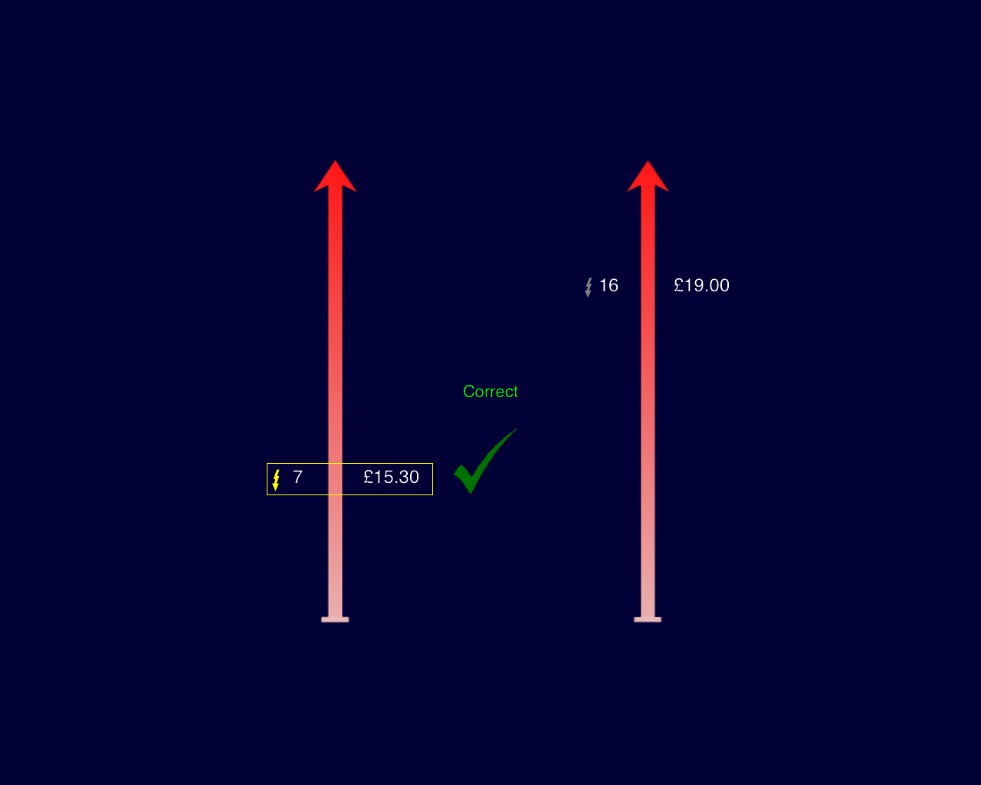
*

*If you are incorrect, the correct option will become highlighted:*

*
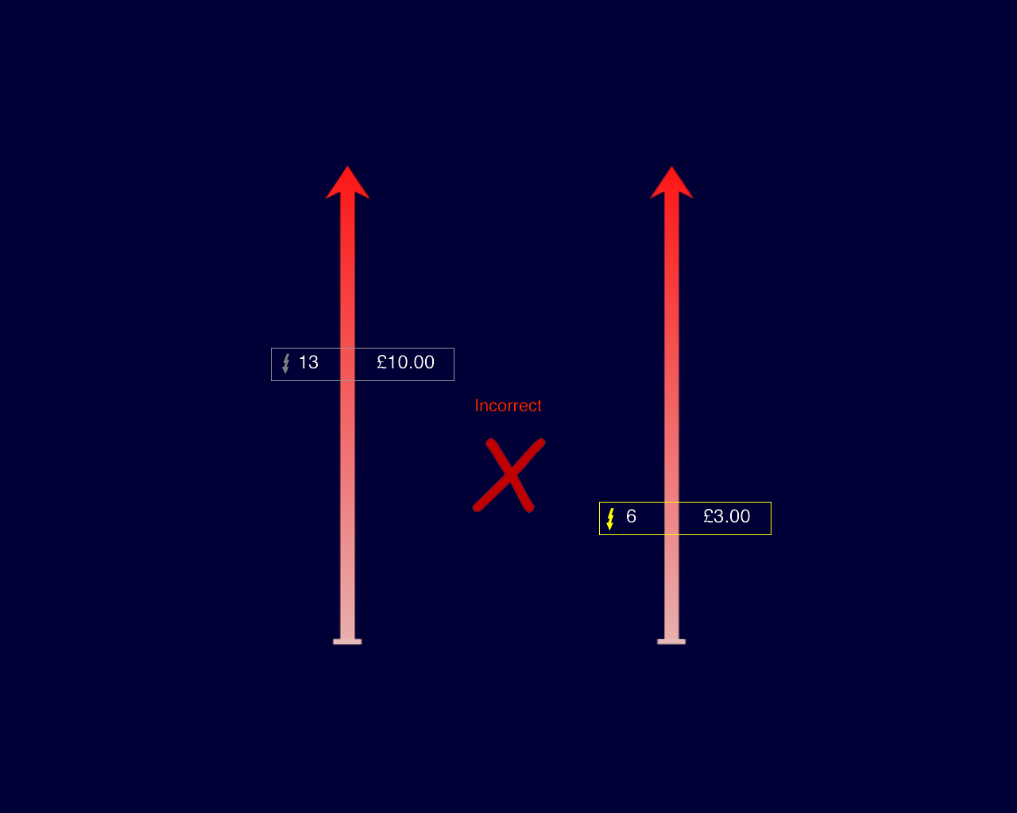
*

*Every few trials, we would like you to indicate your general impression of MJ. To indicate your impression, please use the mouse and respond on a scale from nasty to nice. Here’s what the screens will look like:*

*
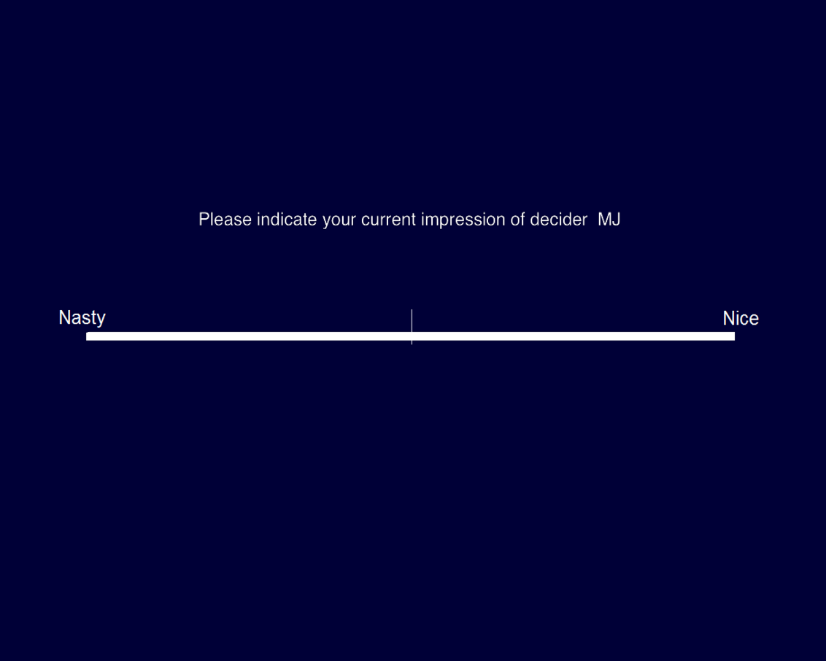
*

*We would also like you to indicate how certain or uncertain you are about your impressions. To indicate your certainty, please use the mouse and respond on a scale from very uncertain to very certain. Here’s what the screens will look like:*

*
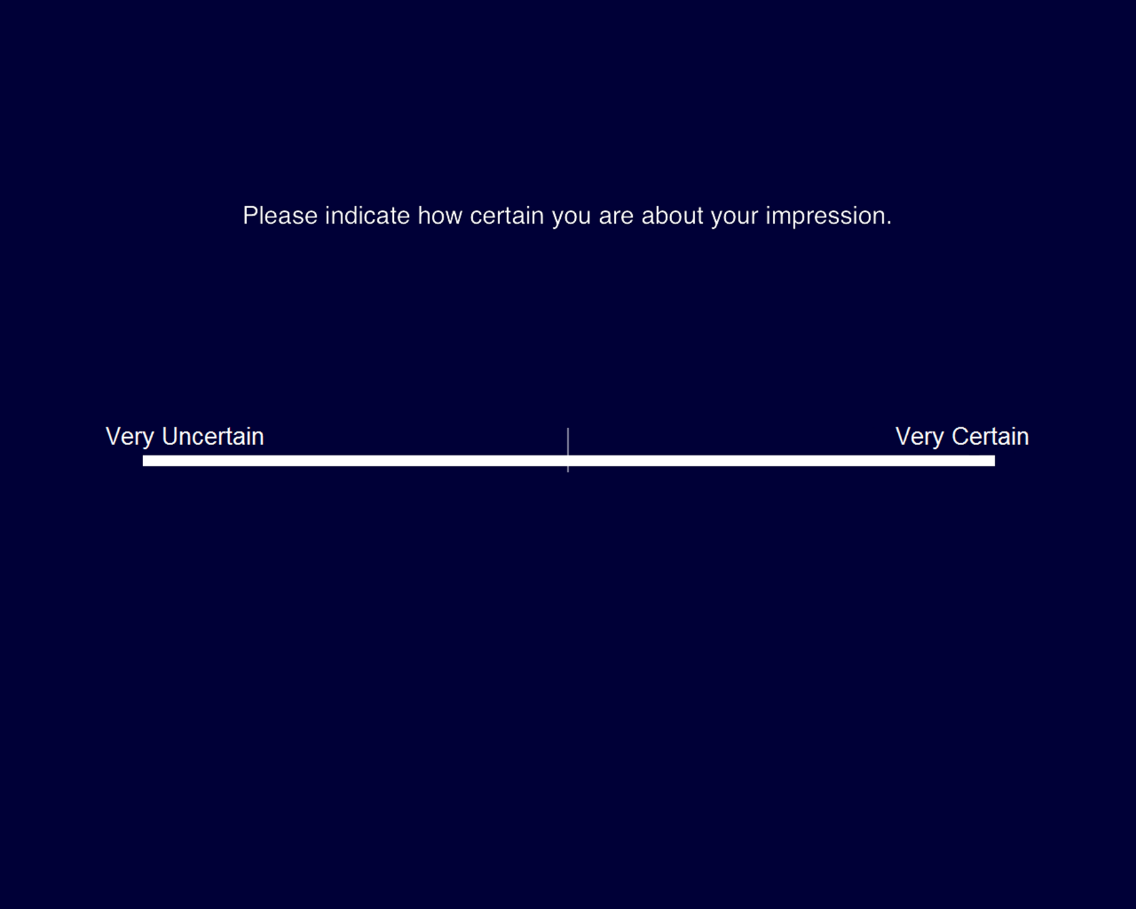
*

*The Prediction Task will last approximately 20 minutes.*

*When you have finished the Prediction Task, we will ask you to complete a few additional tasks. The instructions for these will be provided on the computer screen. If you have any questions at any time, please notify the experimenter using the buzzer.*

- 1. **Post-task and debriefing questions**.

Immediately after the second decision stage, the participants responded to the following questions on a continuous visual analog scale ranging from 0 (not at all) – 100 (very much):

- **Morality and competence of the peer**
- How kind do you think SR/MJ is?
- How selfish do you think SR/MJ is?
- How moral do you think SR/MJ is?
- How intelligent do you think SR/MJ is?
- How competent do you think SR/MJ is?
- **Attitude toward the peer**
- How much do you like SR/MJ?
- How much do you admire SR/MJ?
- **Perceived similarity with the peer and perceived changes in decision-making**
- How similar do you feel to SR/MJ?
- During the experiment, you made decisions before and after observing SR/MJ, who has previously faced similar choices. To what extent did you choose differently after you observed SR/MJ compared with before?
- **Emotional responses**
- How much did SR’s/MJ’s choices make you feel uneasy?
- How much did SR’s/MJ’s choices make you feel proud about your own choices?
- How much did SR’s/MJ’s choices make you feel regretful about your own choices?
- How much did SR’s/MJ’s choices make you feel ashamed about your own choices?
- How much did SR’s/MJ’s choices make you feel embarrassed about your own choices?

We combined ratings of competence and intelligence into a “competence” measure (Cronbach’s α = 0.89); ratings of kindness, morality, and (reversed) selfishness into a “morality” measure (Cronbach’s α = 0.93); and ratings of liking and admiration into an “attitude” measure (Cronbach’s α = 0.80).

After these questions, the participants filled out a debriefing and manipulation check questionnaire online. These include: 1) to what extent they and the receiver found the ‘level 8’ shock aversive; 2) to what extent they felt their decisions were being observed; 3) to what extent the experimenter has made the instruction clear; 4) what gender they thought the receiver was and whether such thoughts influenced their behavior; 5) whether they knew about the Milgram experiments and if so, whether such knowledge had influenced their behaviors; 6) what do they think was/were the goal(s) of the present study. The two groups did not differ on any of these dimesons (see **Table S1** and sections 2.1 and 2.2 below).

1. **Supplementary Results of Study 2**
   1. **Post-task manipulation checks**

Consistent with the impression ratings during the prediction stage, post-task manipulation check ratings indicated that the prosocial peer was perceived as more moral (Mann-Whitney *U* test: *z = -*6.76, *P* < 0.001, **Fig. S1b**) but only marginally more competent (Mann-Whitney *U* test: *z = -*1.95, *P* = 0.052, **Fig. S1c**) than the antisocial peer. Attitudes toward the prosocial peer were significantly more favorable than attitudes toward the antisocial peer (Mann-Whitney *U* test: *z = -*4.96, *P* < 0.001, **Fig. S1d**). Patterns of emotion ratings can be found in **Supplementary Figure 1e**.

- 1. **Results of the debriefing questions**

To rule out concern for reputation and reciprocity, we maintained anonymity throughout the experiment session. We collected three measures in a debriefing questionnaire to help support our claim that our results are not likely to be driven by concerns about reputation and reciprocity. The first item measured subjects’ beliefs that their identity would not be revealed to the receiver. Participants responded on a scale from 1 (fully believe) to 5 (do not at all believe). Over the entire population, participants’ ratings indicate they fully believed their identity would be kept anonymous (mean rating = 1.24, 95% CI [1.08 1.39]). The two groups did not differ in this respect (Mann-Whitney *U*, *z* = -1.32, *P* = 0.185).

The second item measured participants’ beliefs that their decisions would be kept confidential. Participants responded on a scale from 1 (fully believe) to 5 (do not at all believe). Over the entire population, participants’ ratings indicate they fully believed their decision would be kept confidential (mean rating = 1.31, 95% CI [1.14 1.48]). The two groups did not differ in this respect (Mann-Whitney *U* test, *z* = -0.92, *P* = 0.357).

The third item measured participants’ subjective feelings that their decisions were “observed” during the experiment. Participants responded on a scale from 1 (never observed) to 8 (always observed). Over the entire population, participants’ ratings indicate a low-to-moderate level of feeling observed (mean rating = 3.38, 95% CI [2.89 3.88]). The two groups did not differ in this respect (Mann-Whitney *U*, *z* = -0.40, *P* = 0.685).

Additionally, we checked whether the participants considered the electric shocks to be unpleasant to themselves and to the receiver. Participants evaluated the unpleasantness of the shocks to themselves and to the receiver on a scale from 1 (not unpleasant at all) to 7 (extremely unpleasant). Overall, unpleasantness for self (mean rating = 5.13, 95% CI [4.87 5.39]) and for the receiver (mean rating = 5.58, 95% CI [5.38 5.80]) were moderate-to-high. The two groups did not differ in these respects (Mann-Whitney *U* test for self unpleasantness *z* = -0.65, *P*= 0.519; for receiver unpleasantness, *z* = -1.43, *P* = 0.153).

To estimate whether awareness of the goal of our study influence participants’ behavior, we included an open-ended question in the debriefing questionnaire “What do you think was/were the goal(s) of the present study?”. Responses that mentioned behavioral changes were counted as aware of the goal of our study. Six from the antisocial group and seven from the prosocial group were aware of our goal, which did not differ significantly (χ^2^ = 0, *P* = 1). Statistically controlling for this self-reported awareness of the study goal did not affect the findings concerning the changes in harm aversion and in money- and pain-driven value accumulation (see Section 2.8 **Estimating the impact of the awareness of the goals of the study** below).

For the role assignment procedure, we wanted to make sure that the participants did not know the gender of the other participant. In the debriefing questionnaire, we asked participants “What gender do you think the Receiver was?”, where they could respond with “male”, “female” and “not sure”. If they indicated a gender, then we asked a follow-up question “How much do you think the suspected gender of the receiver influenced your decisions?”, where they could respond on a scale from 1 (not at all) to 5 (very much). Over the entire population, participants’ ratings on the second question indicated that they were not influenced by the suspected gender of the receiver (mean rating = 1.31, 95% CI [1.11 1.51]). The two groups did not differ in this respect (Mann-Whitney *U* test, *z* = -0.92, *P*= 0.358).

- 1. **Analysis of the harm aversion parameter**.

To examine the changes of harm aversion across the two decision stages for the two groups, we carried out a Friedman Rand Test (Beasley & Zumbo, 2003) with group (prosocial vs. antisocial) as the between-subject factor and stage (baseline vs. post-influence) as the within-subject factor. We only observed a significant interaction between group and stage on harm aversion (*F* (1, 66) = 41.23, *P* < 0.001). Neither the main effect of group (*F* (1, 66) < 0.001) nor the main effect of stage (*F* (1, 66) = 0.84) was significant. The difference in harm aversion between the prosocial and antisocial groups was not significant before the prediction stage (Mann-Whitney *U* test: *z* = -0.90, *P* = 0.371), but after the prediction stage the prosocial group was marginally more harm averse overall than the antisocial group (Mann-Whitney *U* test: *z* = -1.77, *P* = 0.08).

- 1. **Excluding participants with extreme harm aversion in the baseline stage.**

To rule out the potential confound of “regression-to-the-mean” due to the exclusion criterion reported in the main text, we applied a symmetric exclusion criterion to both groups, namely, excluding participants from both groups whose harm aversion in the baseline was either lower than 0.2 or higher than 0.8, leaving 28 participants in each group. For this subset of participants, we observed a significant interaction between group (prosocial vs. antisocial) and stage (baseline vs. post-influence decision stage) on harm aversion (*F* (1, 54) = 71.30, *P* < 0.001). Specifically, the harm aversion for the prosocial group significantly increased from the baseline to post-influence decision stage (κ_1_ = 0.51±0.18, κ_2_ = 0.60±0.22; Wilcoxon signed-rank test: *z* = -3.56, *P* < 0.001), whereas the harm aversion for the antisocial group decreased from the baseline to post-influence decision stage (κ_1_ = 0.43±0.15, κ_2_ = 0.32±0.14; Wilcoxon signed-rank test: *z* = 4.38, *P* < 0.001).

- 1. **Distribution of reaction times**

Due to the heavy-tailed distribution of reaction times (Fig. S2), we log-transformed the raw reaction times data. We then ran a linear mixed-effects models to examine whether reaction times differed between the two group and across the two decision stages. Specifically, group, stage (i.e., baseline vs. post-learning), and their interaction were included as fixed effect, and stage was included as random slope nested on participant. The only significant effect was stage (*B* = -0.35±0.02, CI = [-0.38, -0.31], *t* = -18.94, *P* < 0.001), indicating that reaction times were shorter in the post-learning stage than in the baseline stage.

- 1. **Replicating the effects of objective similarity on the influence effects on drift weights**

Here, we use two alternative analyses to replicate the results we reported in Section 3.2.5 in the main text.

*Analysis 1: Using the Full Trace of the Model Posteriors*

In this first alternative analysis, we looped over all the samples in the trace generated from the 10,000 iterations of the hierarchical model estimation, and ran a frequentist-based linear regression 10,000 times. Then we plotted the distribution of the regression coefficients of interest and examine whether the 95% CI of these coefficients contained 0.

Specifically, the models we ran were:

Model 1: lm(Δw_pain_ ~ objective similarity * peer + prediction accuracy)

Model 2: lm(Δw_money_ ~ objective similarity * peer + prediction accuracy)

As can be seen from the figure below (**Fig. S5**, panels **a** and **d**), the interaction terms are significant for both models (Model 1: CI = [0.047, 0.121]; Model 2: CI = [-0.080, -0.015]). Looking at the effect of objective similarity separately for each group, we found that for the prosocial group, only the association between objective similarity and Δw_pain_ was significant (**Fig. S5**, panel **b** and **e)**. However, for the antisocial group, the associations between objective similarity and both Δw_money_ and Δw_pain_ were significant, although in opposite directions (**Fig. S5**, panel **c** and **f**). The negative effect shown in panel **c** indicates that the participants whose preference were more similar to the antisocial peer not only exhibited more enhanced money-driven value accumulation, but also exhibited larger decrease in pain-driven value accumulation. However, this effect was not observed in analysis we reported in Section 3.2.5 of the main text and was marginally significant in Analysis 2 (see below). Therefore, the evidence for the latter effect (**Fig. S5**, panel c) is not strong and robust.

*Analysis 2*: *Comparing correlation coefficients between groups*

According to a methodology paper (Katahira, 2016), correlations between hierarchical models and non-hierarchical measures (e.g., objective similarity in this study) are recommended. Therefore, we ran partial correlations between objective similarity, on one hand, and ΔW_money_ and Δw_pain_, on the other hand, using partial correlation package in R (‘ppcor’), and compare the correlation coefficient between groups using Fisher’s z-transformation. As can be seen from **Table S4**, after controlling for the prediction accuracy in the learning stage (as we did in the regression analyses), Δw_money_ was only significantly correlated with the objective similarity for the antisocial group, whereas Δw_pain_ was only significantly correlated with the objective similarity for the prosocial group. The difference between the correlation coefficients between groups were significant for both Δw_money_ and Δw_pain_. This, again, is consistent with the results we reported in Section 3.2.5 of the main text.

- 1. **Relations between subjective similarity and changes in weights of value accumulation controlling for objective similarity.**

We ran two sets of regression models differing in the way of controlling for objective similarity:

*Objective similarity as a main effect only*

stan_glm(Δw_pain_ ~ subjective similarity * peer + objective similarity + prediction accuracy)

stan_glm(Δw_money_ ~ subjective similarity * peer + objective similarity + prediction accuracy)

*Objective similarity as both a main effect and an interaction*

stan_glm(Δw_pain_ ~ subjective similarity * peer + objective similarity * peer + prediction accuracy)

stan_glm(Δw_money_ ~ subjective similarity * peer + objective similarity * peer + prediction accuracy)

There is no strong evidence favoring the model with the interaction term of “objective similarity * peer” over the model with objective similarity only as a main effect (for the Δw_pain_ models, the expected log predicted density is 0.2±1.8, suggesting the model with the interaction term performed slightly worse in fitting the data; for the Δw_money_ model, the expected log predicted density is -1.1±0.5). Therefore, we reported the results based on the model where objective similarity was included only as a main effect.

Controlling participants’ objective similarity with the peers, the interactions between subjective similarity and peer were still significant (for w_pain_, *B* = 0.064±0.023, credible interval = [0.028, 0.100]; for w_money_, *B* = -0.049±0.019, credible interval = [-0.077, -0.017]). Specifically, changes in pain-driven value accumulation (Δw_pain_) were positively associated with subjective similarity with the prosocial peer, but not the antisocial peer (prosocial peer, *B* = 0.049±0.019, credible interval = [0.019, 0.078]; antisocial peer, *B* = -0.017±0.018, credible interval = [-0.046, 0.011]). Similarly, changes in money-driven value accumulation (Δw_money_) were negatively associated with subjective similarity with the prosocial peer, but not with the antisocial peer (prosocial peer, *B* = -0.055±0.015, credible interval = [-0.079, -0.030]; antisocial peer, *B* = -0.006±0.015, credible interval = [-0.030, 0.017]).

- 1. **Estimating the impact of the awareness of the goals of the study.**

Removing the participants who were aware of the goals of the study (6 from the antisocial group and 7 from the prosocial group) from data analysis did not change the results reported in the main text. Specifically, the interaction between group (prosocial vs. antisocial) and stage (baseline vs. post-influence decision stage) on harm aversion (*F* (1, 53) = 36.28, *P* < 0.001). Specifically, the harm aversion for the prosocial group significantly increased from the baseline to post- influence decision stage (κ_1_ = 0.44±0.24, κ_2_ = 0.52±0.29; Wilcoxon signed-rank test: z = -2.94, *P* = 0.03), whereas the harm aversion for the antisocial group decreased from the baseline to post-influence decision stage (κ_1_ = 0.53±0.22, κ_2_ = 0.44±0.26; Wilcoxon signed- rank test: z = -3.54, *P* < 0.001).

Next, we examined the influence effects on the money- and pain-driven value accumulation across after controlling for self-reported aware of the goals of the study. For the regression that examined the changes in money-driven value accumulation (Δw_money_), the interaction between peer (prosocial vs. antisocial) and stage (baseline vs. post-influence decision stage) was significant (*B* = -0.037±0.023, credible interval = [-0.074, -0.000]). Specifically, the association between objective similarity and Δw_money_ was only significant for the antisocial group (*B* = 0.033±0.017, credible interval = [0.008, 0.062]), but not for the prosocial group (*B* = -0.005±0.016, credible interval = [-0.030, 0.020]). For the regression that examined the pain-driven value accumulation (Δw_pain_), the interaction between group (prosocial vs. antisocial) and stage (baseline vs. post-influence decision stage) was also significant (*B* = 0.077±0.029, credible interval = [0.033, 0.123]). Specifically, the association between objective similarity and Δw_pain_ was only significant for the prosocial group (*B* = 0.056±0.020, credible interval = [0.025, 0.088]), but not for the antisocial group (*B* = -0.022±0.021, credible interval = [-0.055, 0.011]).

1. **Supplementary Tables**

**Table S1. Prosocial and antisocial groups are matched across demographic and personality traits**

| Item | Prosocial group | Antisocial group | *Z* | *p* |
| --- | --- | --- | --- | --- |
|  |  |  |  |  |
| Age (years) | 22.5±3.8 | 23.0±4.7 | 0.06 | 0.95 |
| Education | 2.7±1.2 | 2.3±1.3 | 1.42 | 0.16 |
| Income | 2.4±1.3 | 2.6±1.5 | 0.40 | 0.69 |
| Religiosity | 1.9±1.3 | 2.1±1.5 | 0.30 | 0.77 |
| Empathic concern | 19.1±3.8 | 18.3±4.5 | 0.86 | 0.39 |
| Callous affect | 44.3±12.7 | 44.4±13.2 | 0.03 | 0.98 |
| Utilitarianism | 4.1±0.6 | 3.9±0.8 | 1.11 | 0.27 |
|  |  |  |  |  |

*Notes: Education was assessed by a 5-point scale, ranging from “High school/A-Levels” to “Postgraduate/Professional degree or other”. Income (total amount of income earned in the year 2015) was assessed by a 10-point scale ranging from “Under £5,000” to “Over £100,000”. Z: z-value of Mann-Whitney U test.*

**Table S2. Posterior Predictive Checks for the preferred model**

| Summary statistics | Observed | Predicted | SD of predicted | Credible | Quantile | Mahalanobis |  |
| --- | --- | --- | --- | --- | --- | --- | --- |
| **Prosocial group** |  |  |  |  |  |  |  |
| % of harmful decision | 0.52 | 0.52 | 0.23 | TRUE | 57.38 | 0.01 |  |
| Mean harmful RT | 3.25 | 3.69 | 1.20 | TRUE | 38.12 | 0.23 |  |
| 10q of harmful RT | 1.20 | 1.60 | 0.51 | TRUE | 19.63 | 0.79 |  |
| 30q of harmful RT | 1.86 | 2.15 | 0.71 | TRUE | 36.74 | 0.41 |  |
| 50q of harmful RT | 2.47 | 2.85 | 0.99 | TRUE | 37.42 | 0.39 |  |
| 70q of harmful RT | 3.56 | 3.98 | 1.41 | TRUE | 41.99 | 0.30 |  |
| 90q of harmful RT | 5.94 | 6.70 | 2.43 | TRUE | 41.78 | 0.31 |  |
|  |  |  |  |  |  |  |  |
| Mean helpful RT | 3.45 | 3.97 | 1.35 | TRUE | 57.61 | 0.39 |  |
| 10q of helpful RT | 1.41 | 1.76 | 0.67 | TRUE | 30.89 | 0.52 |  |
| 30q of helpful RT | 2.02 | 2.38 | 0.89 | TRUE | 39.90 | 0.40 |  |
| 50q of helpful RT | 2.71 | 3.15 | 1.18 | TRUE | 42.51 | 0.38 |  |
| 70q of helpful RT | 3.72 | 4.36 | 1.62 | TRUE | 41.89 | 0.39 |  |
| 90q of helpful RT | 6.34 | 7.07 | 2.63 | TRUE | 46.04 | 0.28 | |
|  |  |  |  |  |  |  | |
| **Antisocial group** |  |  |  |  |  |  | |
| % of harmful decision | 0.54 | 0.54 | 0.20 | TRUE | 47.78 | 0.04 | |
| Mean harmful RT | 3.52 | 3.86 | 1.51 | TRUE | 51.73 | 0.22 | |
| 10q of harmful RT | 1.29 | 1.59 | 0.57 | TRUE | 36.62 | 0.52 | |
| 30q of harmful RT | 1.85 | 2.17 | 0.84 | TRUE | 46.41 | 0.38 | |
| 50q of harmful RT | 2.61 | 2.93 | 1.21 | TRUE | 50.42 | 0.27 | |
| 70q of harmful RT | 3.81 | 4.18 | 1.75 | TRUE | 52.39 | 0.21 | |
| 90q of harmful RT | 6.82 | 7.17 | 3.07 | TRUE | 55.70 | 0.11 | |
|  |  |  |  |  |  |  | |
| Mean helpful RT | 3.85 | 4.44 | 1.72 | TRUE | 54.44 | 0.35 | |
| 10q of helpful RT | 1.48 | 1.85 | 0.66 | TRUE | 30.69 | 0.57 | |
| 30q of helpful RT | 2.17 | 2.59 | 0.98 | TRUE | 40.46 | 0.42 | |
| 50q of helpful RT | 2.93 | 3.50 | 1.40 | TRUE | 42.18 | 0.41 | |
| 70q of helpful RT | 4.08 | 4.92 | 2.03 | TRUE | 41.25 | 0.41 | |
| 90q of helpful RT | 7.39 | 8.01 | 3.45 | TRUE | 51.31 | 0.20 | |

*Notes: 10q ~ 90q: 10^th^ ~ 90^th^ quantile of RT distribution; Credible: whether observed data falls in the 95% credible interval of the simulated data; Mahalanobis: Mahalanobis distance of the observed data from the center of distribution of the simulated data.*

**Table S3. Tests of normality**

| Variable | Shapiro-Wilk (W) | P |
| --- | --- | --- |
| **Prosocial group** |  |  |
| κ (baseline) | 0.94 | 0.044 |
| κ (post-influence) | 0.91 | 0.011 |
| β (baseline) | 0.51 | < 0.001 |
| β (post-influence) | 0.33 | < 0.001 |
| prior impression | 0.64 | < 0.001 |
| perceived similarity | 0.95 | 0.087 |
| perceived shift | 0.90 | 0.005 |
| **Antisocial group** |  |  |
| κ (baseline) | 0.91 | 0.008 |
| κ (post-influence) | 0.88 | 0.001 |
| β (baseline) | 0.58 | < 0.001 |
| β (post-influence) | 0.46 | < 0.001 |
| prior impression | 0.64 | < 0.001 |
| perceived similarity | 0.97 | 0.538 |
| perceived shift | 0.91 | 0.007 |

**Table S4. Correlations between objective similarity and changes in pain-driven and money-driven value accumulation**

| Coefficient (r) | ΔW_money_ | ΔW_pain_ |
| --- | --- | --- |
| Objective similarity  (prosocial) | -0.141 | 0.421* |
| Objective similarity  (antisocial) | 0.397* | -0.325^#^ |
| Fisher’s z | 2.17* | 3.10* |

Notes: *: *p* < 0.05, #: 0.05 < *p* < 0.1

1. **Supplementary Figures**


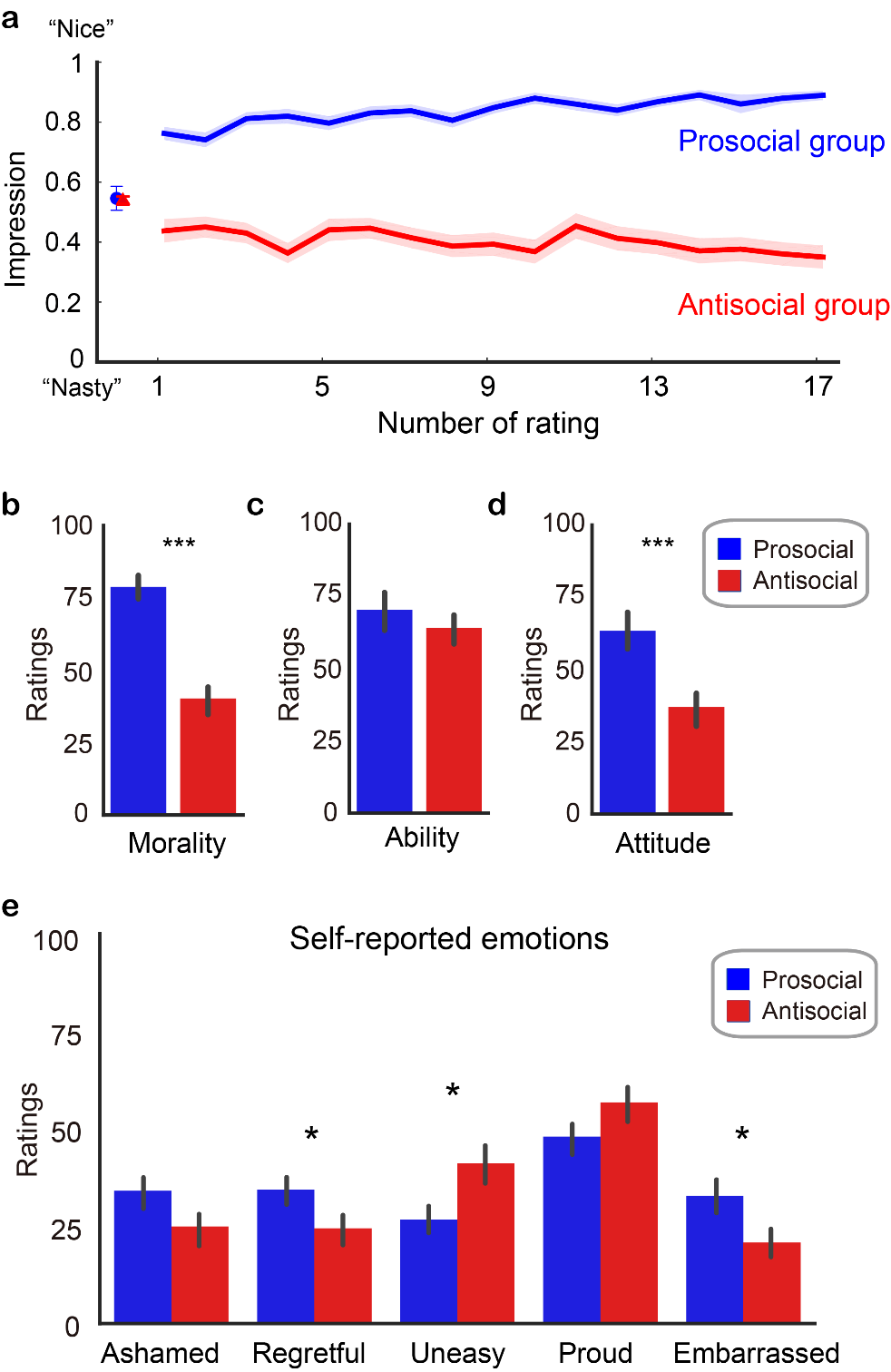


**Supplementary Figure 1**. Subjective impressions of the peers in Study 2. (a) Impressions of the peers across trials during the prediction stage. Initial data points indicate participants’ expectations about the peer’s character before they observed any of their choices (blue for prosocial group and red for antisocial group). (b-d) Ratings of morality, ability, and attitudes toward the peers collected at the end of the experiment. (e) Results of self-reported emotions. Error bars indicate s.e.m. *: *p* < 0.05, ***: *p* < 0.001.

**
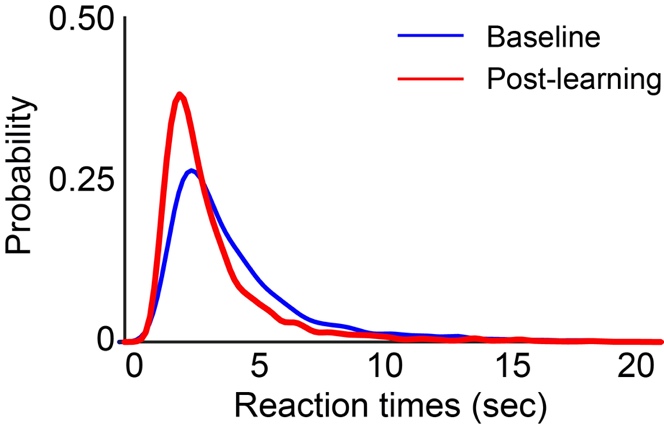
**

**Supplementary Figure 2.** Distribution of reaction times after excluding trials with extreme reaction times.


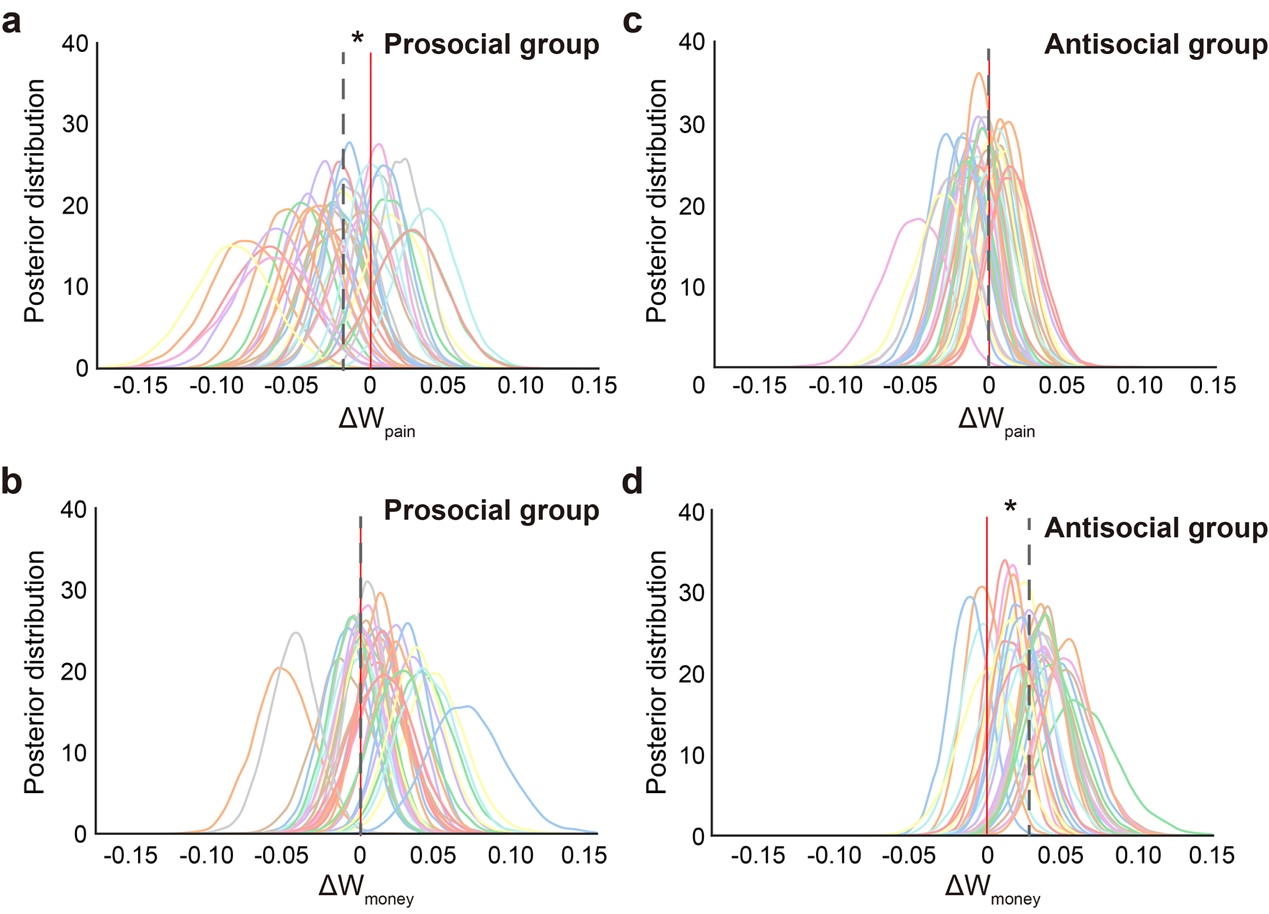


**Supplementary Figure 3.** Individual effects of changes in weights of value accumulation. Changes in pain-driven (a) and money-driven (b) value accumulation for the prosocial group. Changes in pain-driven (c) and money-driven (d) value accumulation for the antisocial group. Dotted line indicates the group-level estimate and the red line indicates 0. * indicates significant group-level effects (i.e., credible interval excludes 0).

**
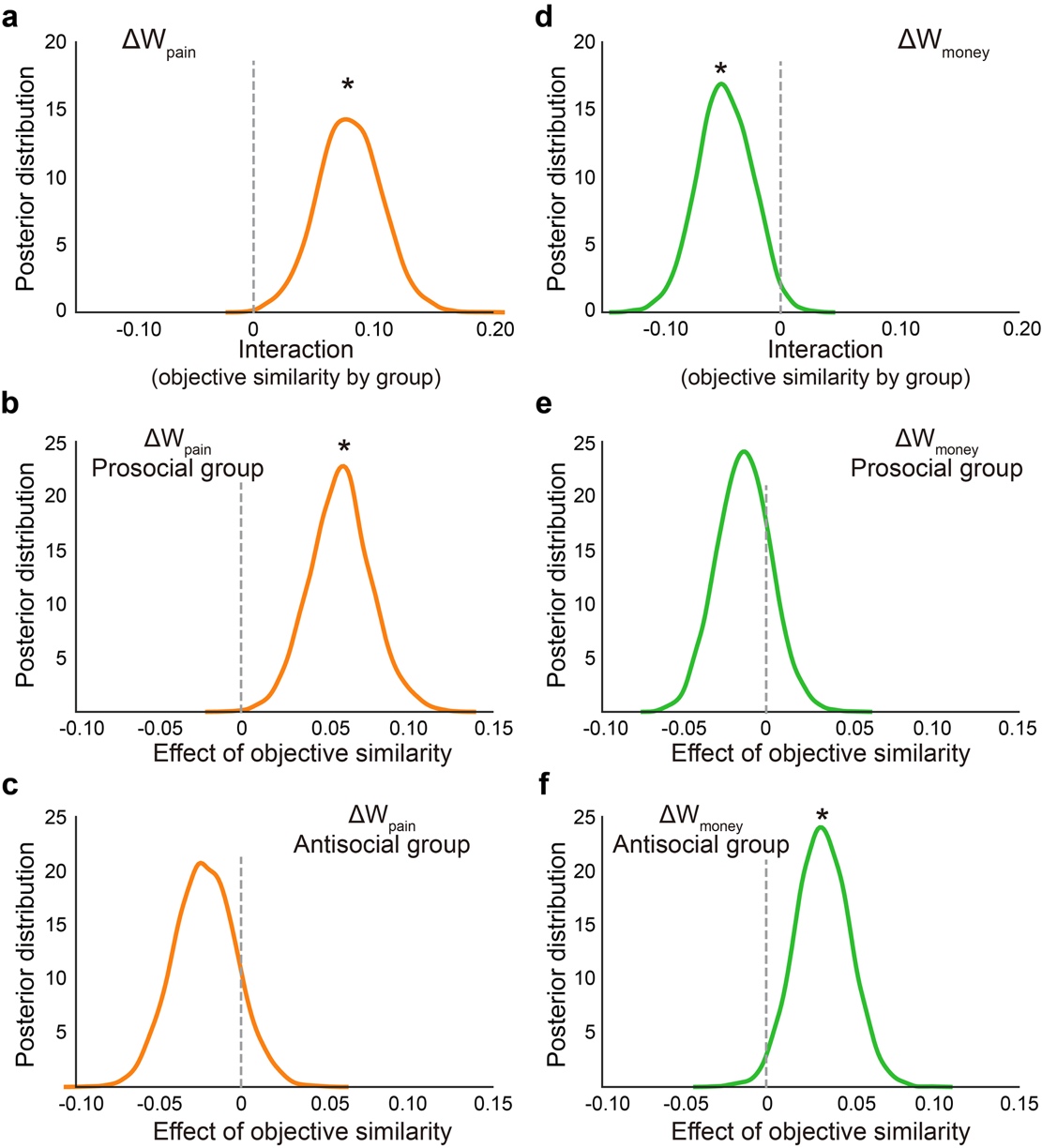
**

**Supplementary Figure 4.** Posterior distribution of coefficients of the Bayesian regression models. (a) The interaction effect of objective similarity and group on Δw_pain_. (b) The effect of objective similarity on Δw_pain_ for the prosocial group. (c) The effect of objective similarity on Δw_pain_ for the antisocial group. (d) The interaction effect of objective similarity and group on Δw_money_. (e) The effect of objective similarity on Δw_money_ for the prosocial group. (f) The effect of objective similarity on Δw_money_ for the antisocial group. * indicates significance (i.e., credible interval excludes 0).

**
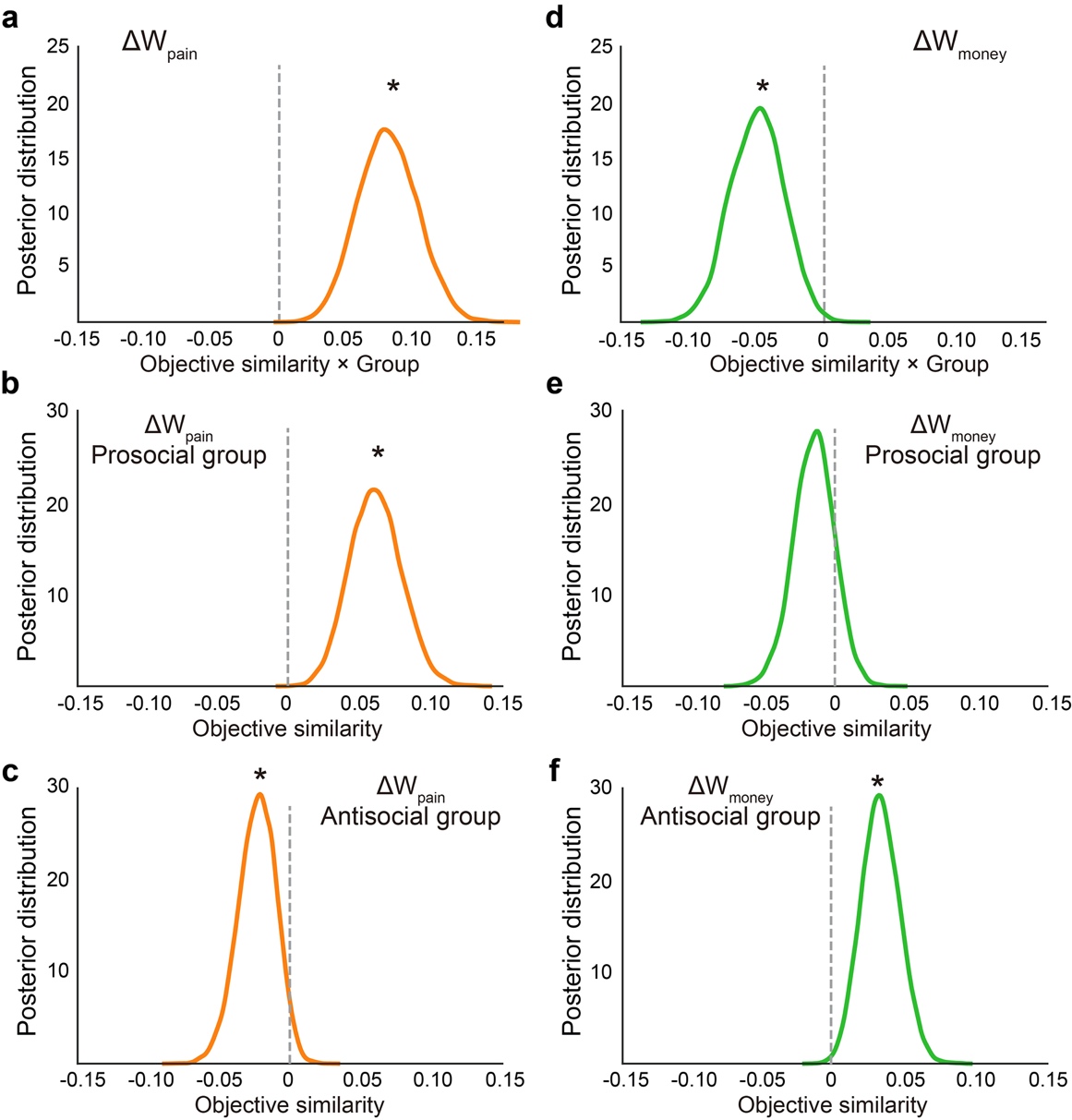
**

**Supplementary Figure 5.** Distribution of coefficients of the frequentist-based linear regression models. (a) The interaction effect of objective similarity and group on Δw_pain_. (b) The effect of objective similarity on Δw_pain_ for the prosocial group. (c) The effect of objective similarity on Δw_pain_ for the antisocial group. (d) The interaction effect of objective similarity and group on Δw_money_. (e) The effect of objective similarity on Δw_money_ for the prosocial group. (f) The effect of objective similarity on Δw_money_ for the antisocial group. * indicates significance (i.e., credible interval excludes 0).

**References**

Beasley, T. M., & Zumbo, B. D. (2003). Comparison of aligned Friedman rank and parametric methods for testing interactions in split-plot designs. *Computational statistics & data analysis*, *42*(4), 569-593.

Katahira, K. (2016). How hierarchical models improve point estimates of model parameters at the individual level. *Journal of Mathematical Psychology*, *73*, 37-58.
